# Supplementary material for: Cellular immune response to SARS-CoV-2 and clinical presentation in individuals exposed to endemic malaria
Source: Cell Rep. 2024 Jul 24;43(8):114533. doi: 10.1016/j.celrep.2024.114533 (PMC11372439; doi:10.1016/j.celrep.2024.114533)
Supplement: Document S1. Figures S1‒S7 [file mmc1.pdf]

Cell Reports, Volume *43*

Supplemental information

Cellular immune response to SARS-CoV-2 and clinical presentation in individuals exposed to endemic malaria

Kesego Tapela, Diana Ahu Prah, Becky Tetteh, Franklin Nuokpem, Daniel Dosoo, Amin Coker, Frederick Kumi-Ansah, Emmanuella Amoako, Kissi Ohene Assah, Charlyne Kilba, Nancy Nyakoe, Darius Quansah, Sylvester Languon, Claudia Adzo Anyigba, Felix Ansah, Seth Agyeman, Irene Amoakoh Owusu, Kristan Schneider, William K. Ampofo, Joe Kimanthi Mutungi, Gloria Amegatcher, Yaw Aniweh, Gordon A. Awandare, Peter K. Quashie, and Yaw Bediako

Supplementary figures

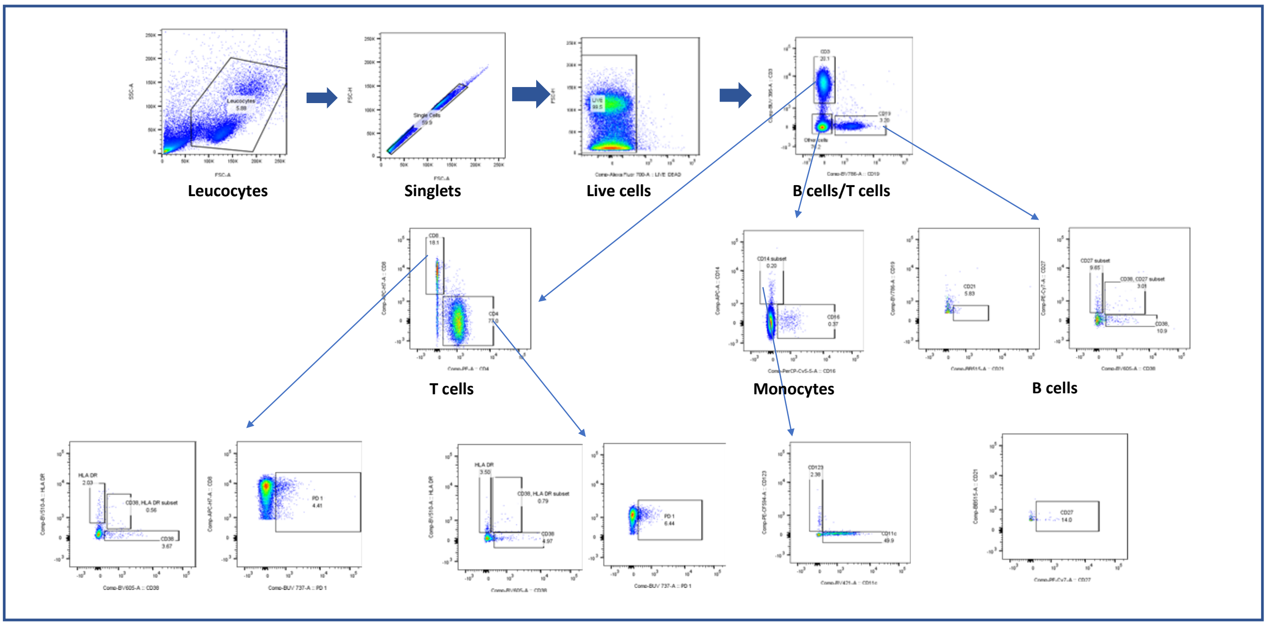

Figure S1: Cell gating for immunophenotyping, Related to Figure 1

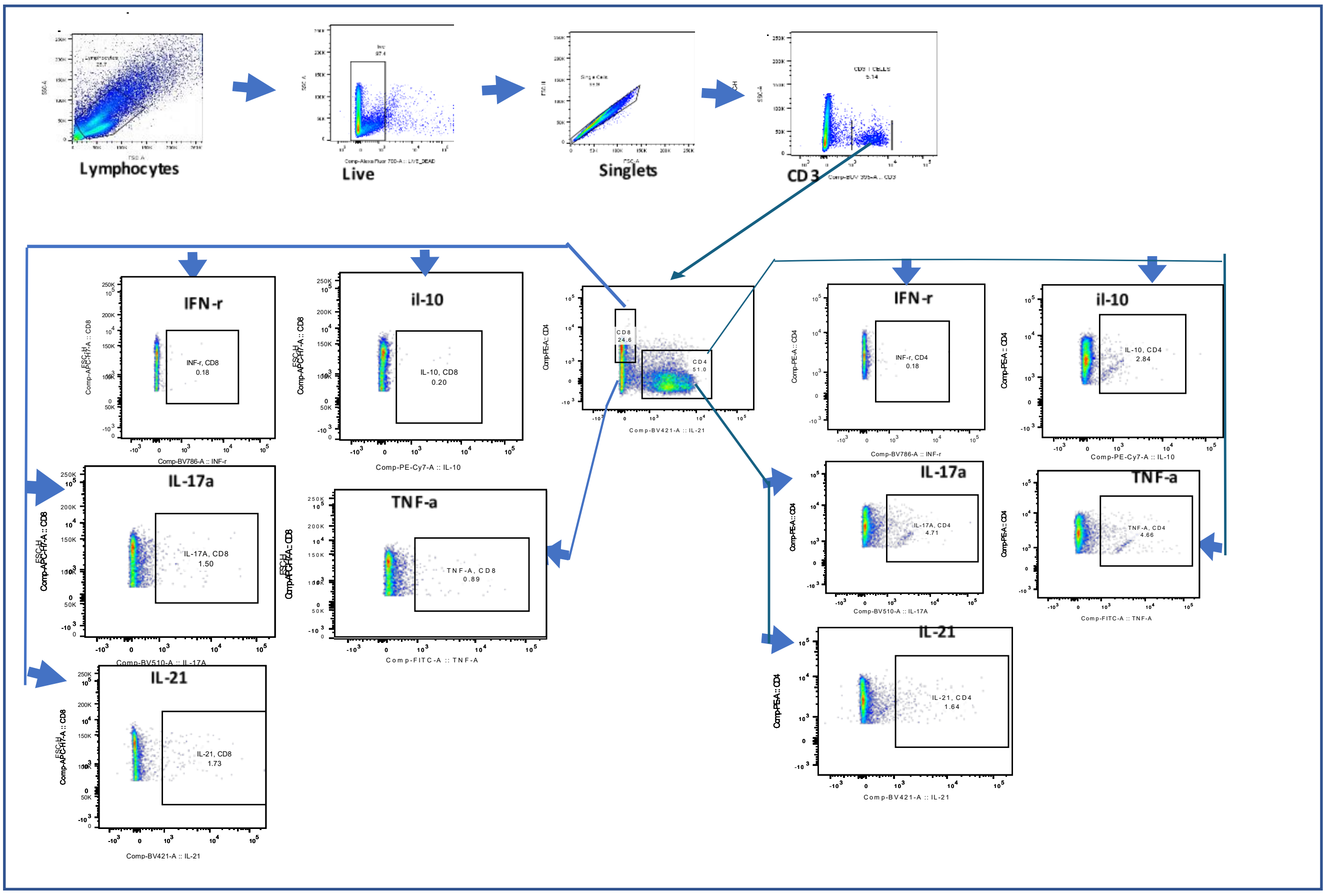

Figure S2: Cell gating for intracellular staining, Related to Figure 2.

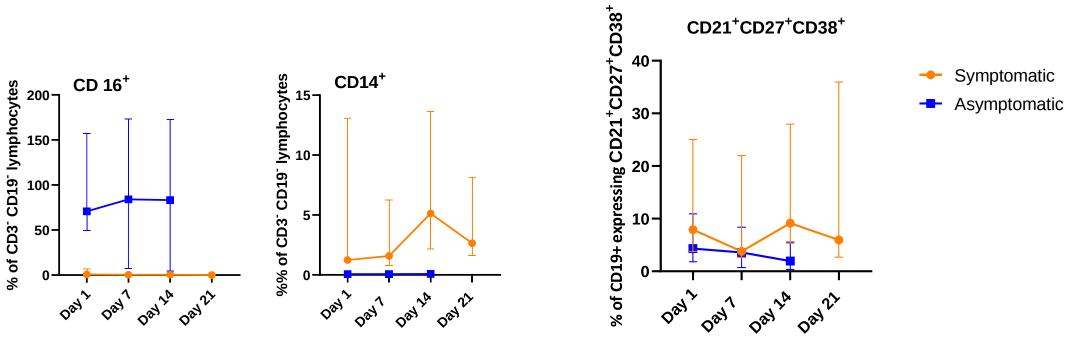

Figure S3: The change in surface cell frequencies over time, Related to Figure 1. The percentage cell proportions were analysed from PBMC of COVID-19 symptomatic (n = 53) and asymptomatic (n =29) patients at different time points for four weeks. The median quantity of the percentage cell proportions for each sampling day is shown by the line graph and the 25th and 75th percentiles are cell percentage proportions.

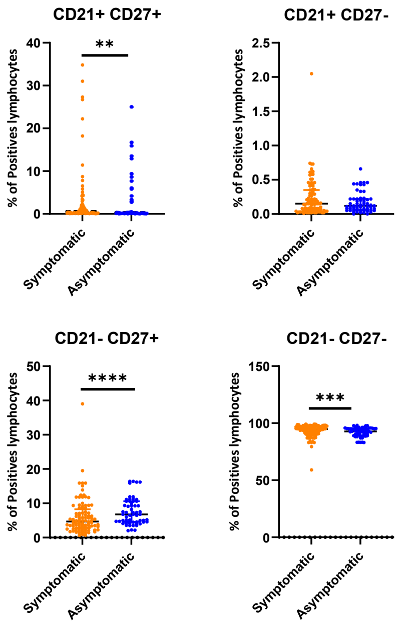

Figure S4: The scatter plots showing the proportion of classical memory B cells ($CD21^{+}CD27^{+}$), naïve B cells ($CD21^{+}CD27^{-}$), atypical memory B cells ($CD21^{-}CD27^{-}$) and activated memory B cells ($CD21^{-}CD27^{+}$), Related to Figure 1. A horizontal line across the scatter plots shows the median baseline samples per participant while the lower and upper dotted lines represent the 1st and 3rd percentiles, respectively. Statistical significance between symptomatic and asymptomatic patients were determined by a two-tailed Mann-Whitney U test (ns: $p > 0.05$, *: $p < 0.05$, **: $p < 0.01$, ****: $p < 0.0001$).

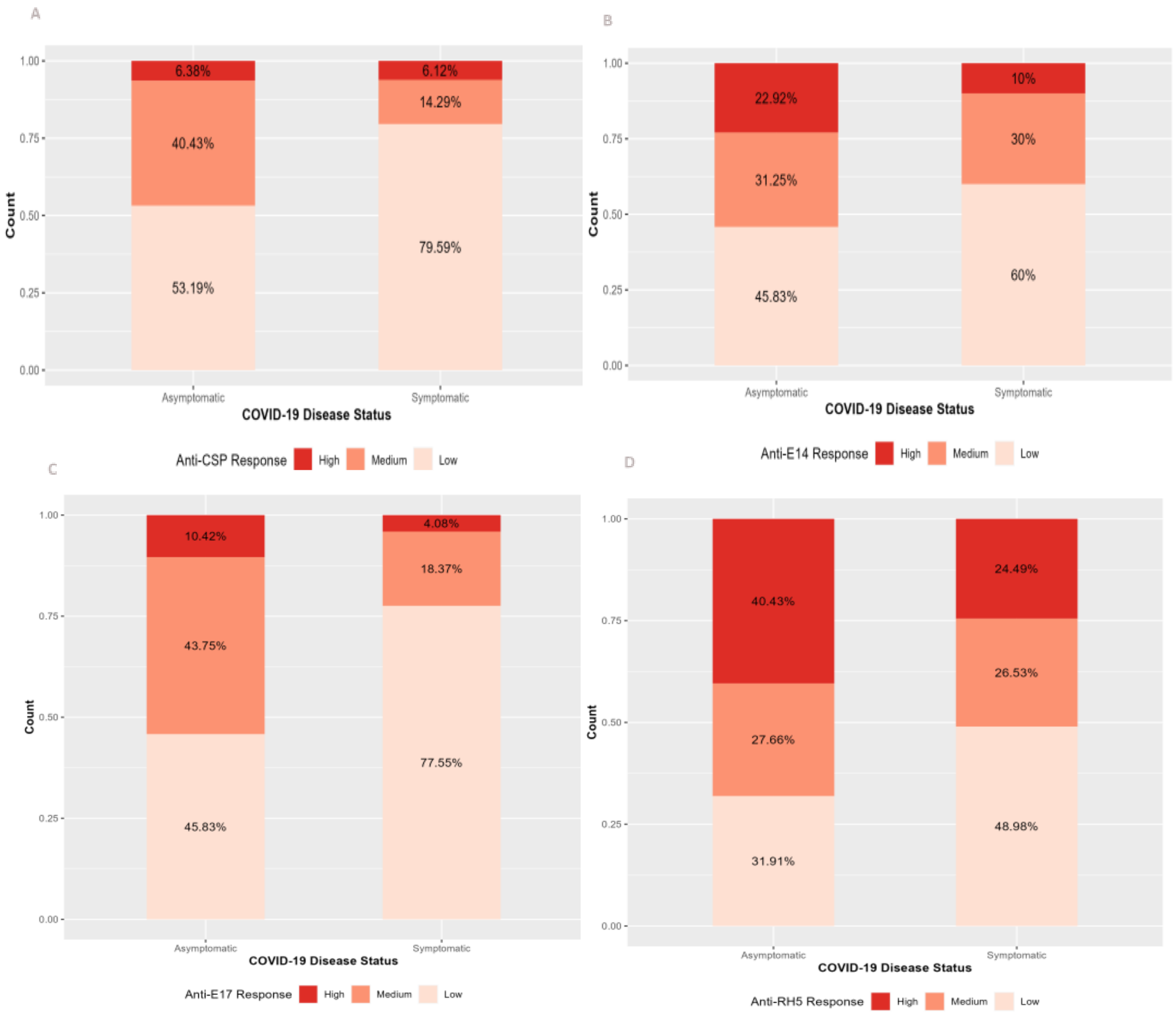

Figure S5: **The stacked bar charts comparing the proportions of antibody levels from (A):PfCSP ,(B):E-14, (C):E-17 and (D):RH5 in symptomatic(n=50) and asymptomatic(n=50) patients, Related to Figure 3.** A finite mixture model was used to segregate the dataset into subpopulations; low, medium, and high antibody responses.

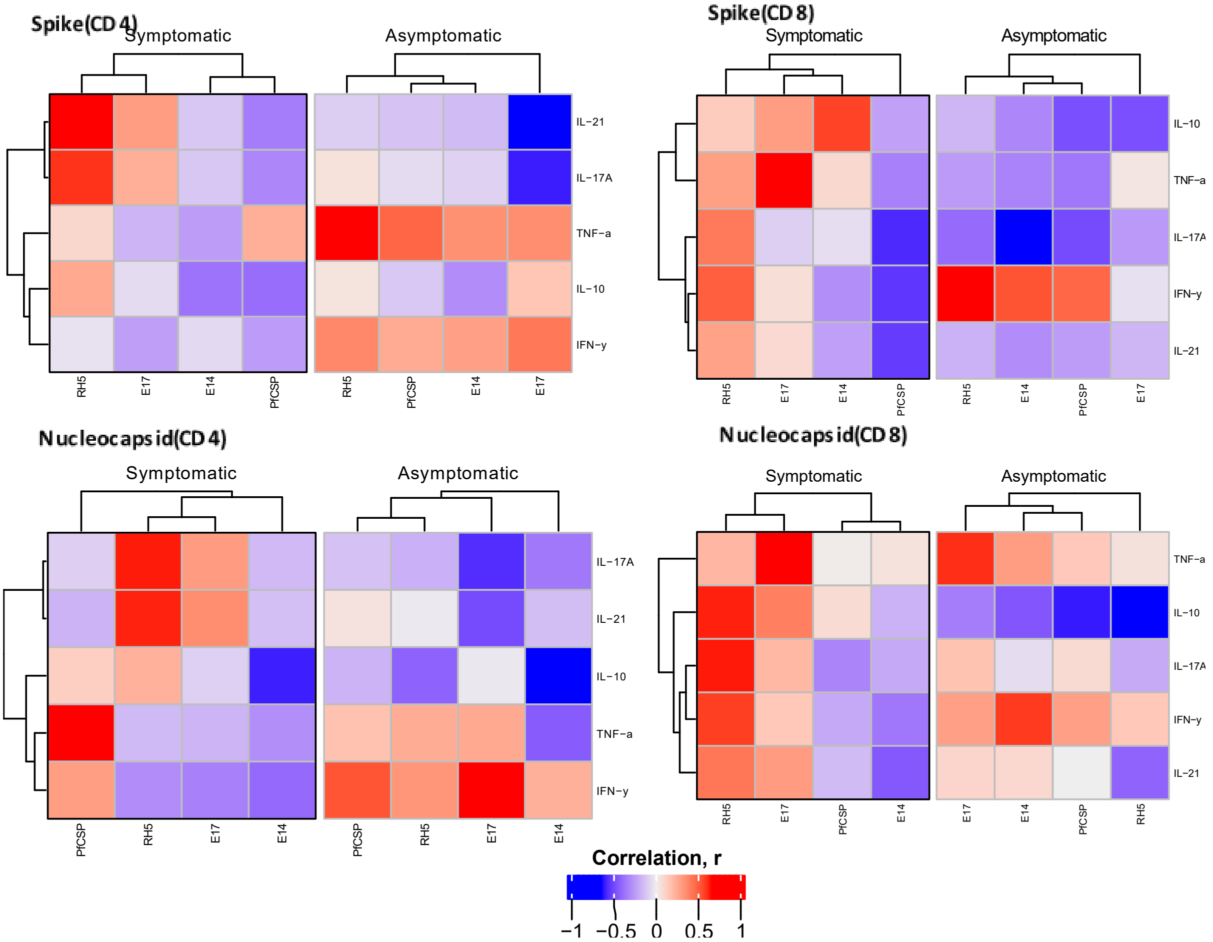

Figure S6:Correlations between anti-*P.falciparum* antibodies and cytokines in symptomatic and asymptomatic COVID-19 individuals, Related to Figures 2 & 3. The cytokine-antibodies clustering patterns are shown in heatmaps and the magnitude of correlation coefficients is shown on a color scale of red (strong positive correlation), white (no correlation) to dark blue (strong negative correlation) signal.

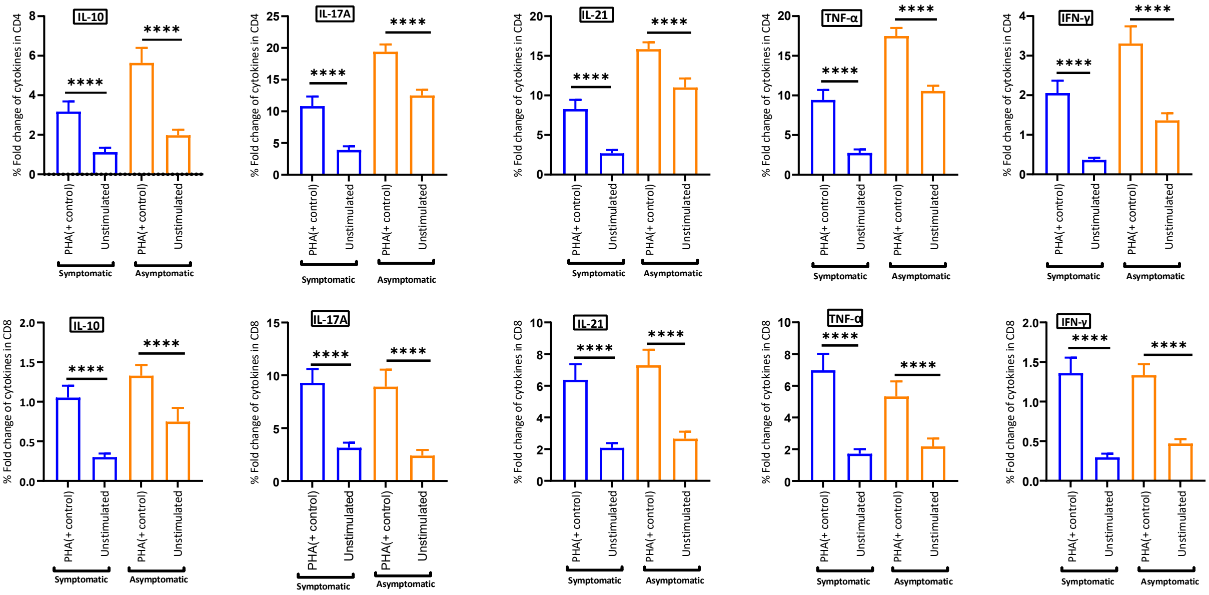

Figure S7: **Comparison of percentage stimulation of cytokines by PHA and unstimulated in symptomatic (n=116) and asymptomatic (n=44) COVID-19 patients, Related to Figure 2.** The bar plots show mean with standard error bars. The percentages were calculated by subtracting the percentages of stimulated from unstimulated cells. Statistical significance was determined by Mann-Whitney U test (ns: $p > 0.05$, *: $p < 0.05$, **: $p < 0.01$, ****: $p < 0.0001$).
