# Supplementary material for: Cellular immune response to SARS-CoV-2 and clinical presentation in individuals exposed to endemic malaria
Source: Cell Rep. 2024 Jul 24;43(8):114533. doi: 10.1016/j.celrep.2024.114533 (PMC11372439; doi:10.1016/j.celrep.2024.114533)
Supplement: Document S2. Article plus supplemental information [file mmc2.pdf]

Cell Reports

Article

Cellular immune response to SARS-CoV-2 and clinical presentation in individuals exposed to endemic malaria

Graphical abstract

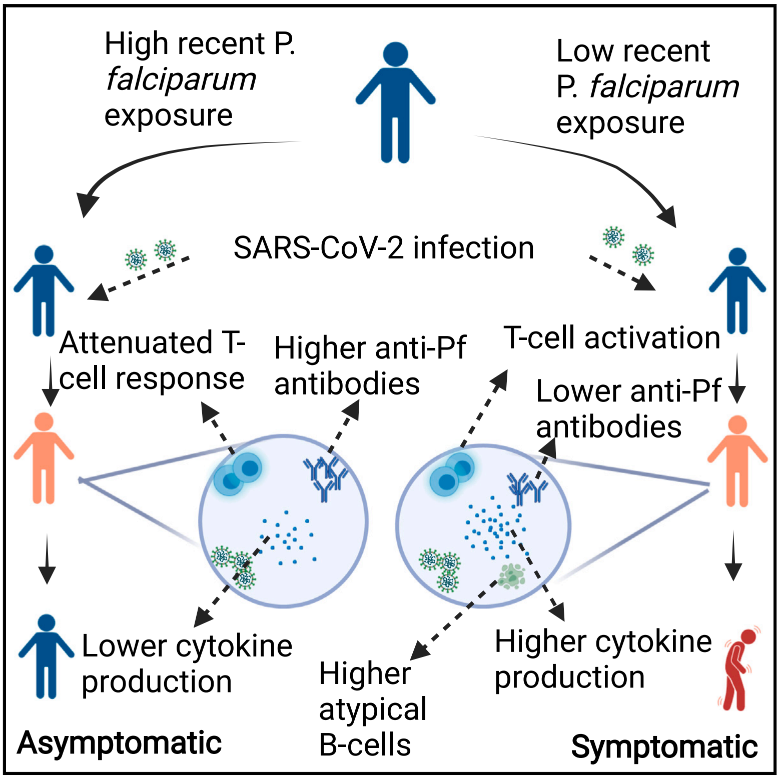

Highlights

- Asymptomatic COVID-19 shows lower T cell response and higher *P. falciparum* antibodies
- Symptomatic COVID-19 shows higher T cell exhaustion and lower *P. falciparum* antibodies
- People with asymptomatic COVID-19 were more likely to show recent exposure to malaria

Authors

Kesego Tapela, Diana Ahu Prah, Becky Tetteh, ..., Gordon A. Awandare, Peter K. Quashie, Yaw Bediako

Correspondence

pquashie@ug.edu.gh (P.K.Q.), ybediako@ug.edu.gh (Y.B.)

In brief

Tapela et al. study the impact of previous malaria exposure on people with COVID-19. Prior malaria exposure may influence the immune response to SARS-CoV-2 and might be linked to milder COVID-19 cases in malaria-endemic regions, offering valuable insights for viral disease management and outbreak control in these populations.

Tapela et al., 2024, Cell Reports *43*, 114533
August 27, 2024 © 2024 The Author(s). Published by Elsevier Inc.
https://doi.org/10.1016/j.celrep.2024.114533

Cell Reports

CellPress
OPEN ACCESS

Article

Cellular immune response to SARS-CoV-2 and clinical presentation in individuals exposed to endemic malaria

Kesego Tapela,[1,2] Diana Ahu Prah,[1] Becky Tetteh,[1,2] Franklin Nuokpem,[1,2] Daniel Dosoo,[1] Amin Coker,[3] Frederick Kumi-Ansah,[4] Emmanuella Amoako,[5,7] Kissi Ohene Assah,[8] Charlyne Kilba,[6] Nancy Nyakoe,[1,2] Darius Quansah,[1,2,8] Sylvester Languon,[1] Claudia Adzo Anyigba,[1,2] Felix Ansah,[1,2] Seth Agyeman,[1,2,7] Irene Amoakoh Owusu,[1] Kristan Schneider,[11] William K. Ampofo,[8] Joe Kimanthi Mutungi,[1] Gloria Amegatcher,[9] Yaw Aniweh,[1,10] Gordon A. Awandare,[1,2] Peter K. Quashie,[1,10,*] and Yaw Bediako[1,7,10,12,*]

[1]West African Centre for Cell Biology of Infectious Pathogens, College of Basic and Applied Sciences, University of Ghana, Legon, Accra, Ghana
[2]Department of Biochemistry, Cell and Molecular Biology, University of Ghana, Legon, Accra, Ghana
[3]Accident and Emergency Unit, The Greater Accra Regional Hospital, Accra, Ghana
[4]Department of Microbiology, Cape Coast Teaching Hospital, Cape Coast, Ghana
[5]Department of Pediatrics, Cape Coast Teaching Hospital, Cape Coast, Ghana
[6]Department of Internal Medicine, Surgery, Pediatrics, and Emergency Medicine, Greater Accra Regional Hospital, Accra, Ghana
[7]Yemaachi Biotech Inc., 222 Swaniker St., Accra, Ghana
[8]Department of Virology, Noguchi Memorial Institute for Medical Research, University of Ghana, Legon, Accra, Ghana
[9]Department of Medical Laboratory Science, School of Biomedical and Allied Sciences, University of Ghana, Accra, Ghana
[10]The Francis Crick Institute, 1 Midland Rd., London NW1 1AT, UK
[11]Department of Mathematics, Hochschule Mittweida, University of Applied Sciences, Mittweida, Germany
[12]Lead contact
*Correspondence: pquashie@ug.edu.gh (P.K.Q.), ybediako@ug.edu.gh (Y.B.)
https://doi.org/10.1016/j.celrep.2024.114533

SUMMARY

Ghana and other parts of West Africa have experienced lower COVID-19 mortality rates than other regions. This phenomenon has been hypothesized to be associated with previous exposure to infections such as malaria. This study investigated the immune response to severe acute respiratory syndrome coronavirus 2 (SARS-CoV-2) and the influence of previous malaria exposure. Blood samples were collected from individuals with asymptomatic or symptomatic COVID-19 (n = 217). A variety of assays were used to characterize the SARS-CoV-2-specific immune response, and malaria exposure was quantified using *Plasmodium falciparum* ELISA. The study found evidence of attenuated immune responses to COVID-19 among asymptomatic individuals, with elevated proportions of non-classical monocytes and greater memory B cell activation. Symptomatic patients displayed higher *P. falciparum*-specific T cell recall immune responses, whereas asymptomatic individuals demonstrated elevated *P. falciparum* antibody levels. Summarily, this study suggests that *P. falciparum* exposure-associated immune modulation may contribute to reduced severity of SARS-CoV-2 infection among people living in malaria-endemic regions.

INTRODUCTION

COVID-19 caused by severe acute respiratory syndrome coronavirus 2 (SARS-CoV-2) was a substantial global threat to humans, with millions of confirmed infections and deaths.[1] The disease's clinical features vary in different individuals and range from asymptomatic to acute respiratory distress syndrome.[2] In Africa, especially West Africa, the reported mortalities remained relatively low[3] for a large part of the pandemic until more virulent variants caused fatalities to rise.[1] Irrespective of the circulating variants,[4,5] reports indicate that most African countries still had a lower incidence of severe infections and death. Moreover, at least 80% of cases appeared to be asymptomatic.[6–8]

It is still not clear why West Africa appears to have had lower COVID-19-related mortality than other parts of the world. However, factors such as a younger population,[9] warm climate,[10] and increased prevalence of infectious diseases, particularly malaria,[11] have been suggested as possible explanations. Malaria, caused by *Plasmodium* parasites, remains one of the world's major health challenges.[12] Malaria caused an estimated number of 247 million cases globally in 2021, of which 96% were reported in tropical sub-Saharan Africa.[12] Malaria and COVID-19 share some pathophysiological characteristics and clinical presentations, such as fever, headache, chills, and sweating, often resulting in misdiagnoses between the two diseases. In both cases, hyperinflammatory responses

This is an open access article under the CC BY license (http://creativecommons.org/licenses/by/4.0/).

Table 1. Characteristics of study participants

	All participants (n = 217)	
	Asymptomatic (n = 62)	Symptomatic (n = 155)
Age[a]		
1–15	–	2
15–45	4	22
>45	3	16
Median age, years (interquartile range [IQR])[a]	41 (21–67)	32 (13–82)
Male	33 (53%)	74 (48%)
Female	29 (47%)	81 (52%)
Median lymphocyte count/μL[a]	–	2.938
SpO2[b]	–	95 (43–100)
Vaccination status		
Vaccinated	–	40
Unvaccinated	62	115
Seropositivity[a]		
Positive	9	48
Negative	31	39

[a]When data were available.
[b]Symptomatic cases only.

and cytokine storms are implicated in symptomatic cases and mortalities.[13,14]

Studies have consistently demonstrated that, following repeated exposure to *Plasmodium* parasites, the host immune system is modulated to reduce inflammation-causing pathology.[15–17] The precise mechanism for such immunomodulation is not well understood; however, there is evidence that the parasite induces epigenetic reprogramming of monocytes, which attenuates the inflammatory response upon re-exposure.[18] Additionally, re-exposure to *Plasmodium* can lead to the suppression of interleukin-12 [IL-12] production by monocytes; the production of IL-12 is linked to severe malaria.[19]

It is possible that repeated exposure to *Plasmodium* parasites may have resulted in the acquisition of immunological tolerance so that affected individuals can effectively regulate inflammatory responses caused by other pathogens. Consequently, this immunological tolerance could help explain why individuals in malaria-endemic settings appear to be protected against severe SARS-CoV-2 infection.[11,20] The observation that COVID-19 mortality within Africa appears to be inversely correlated with malaria endemicity[21,22] provides further anecdotal support for this hypothesis. Although a few studies have explored the link between malaria and COVID-19,[23,24] to date, no proper empirical data have been generated to support malaria-associated protection from severe COVID-19 disease.

Infection with SARS-CoV-2 activates CD8$^+$ and CD4$^+$ T cells. Therefore, T cell lymphopenia in peripheral blood, coupled with increased cell activation and exhaustion, appears to be characteristics of symptomatic COVID-19 cases.[25–28] Similarly, a reduction in B cell frequency has also been associated with symptomatic COVID-19 disease.[29] Studies conducted in asymptomatic individuals have demonstrated slightly elevated T cells, B cells, and monocytes throughout the infection.[29–31] Though informative, most of these studies were conducted in non-African countries and therefore are not representative of malaria-endemic countries.

Focusing on symptomatic and asymptomatic COVID-19 infections, this study aimed to phenotype and characterize antigen-specific T cell responses in Ghanaians with COVID-19 disease and investigate the impact of *Plasmodium* exposure to this response. A better understanding of the cellular immune response to SARS-CoV-2 within the context of malaria endemicity will be useful in guiding better disease management, outbreaks, and potential vaccine design for such populations.

RESULTS

Participant characteristics and sample usage

A total of 217 individuals with confirmed SARS-CoV-2 infection were recruited (Table 1) between January and December 2021. Samples were collected <7 days after symptom onset. Based on available metadata, participants were classified as asymptomatic (n = 62) and symptomatic (n = 155). Cell phenotyping was performed on baseline samples from all 217 individuals and longitudinal samples up to 1 month post diagnosis for 82 individuals (symptomatic n = 53; asymptomatic, n = 29). Cytokine production assays were performed based on the availability of baseline samples (n = 160), comprising both symptomatic (n = 116) and asymptomatic (n = 44) samples. For the anti-malaria antibody response assay, plasma samples (n = 100) comprising symptomatic (n = 50) and asymptomatic (n = 50) samples were used. Peripheral blood mononuclear cell (PBMC) samples (n = 74) obtained from healthy consenting individuals recruited at the University of Ghana in March 2020 who tested negative for SARS-CoV-2 by PCR were used as negative controls.

Asymptomatic SARS-CoV-2-positive individuals display attenuated levels of T cell activation and exhaustion

To evaluate the connection between cellular immune composition and clinical presentation, the study compared the relative frequencies of peripheral blood leukocyte subpopulations in individuals with asymptomatic and symptomatic COVID-19. The ratios of CD4$^+$/CD8$^+$ T cells were higher in symptomatic as compared to asymptomatic individuals, but this difference was not statistically significant (Figure 1A). Moreover, while CD4$^+$ T cell frequencies were comparable between symptomatic and asymptomatic individuals, the frequency of CD8$^+$ T cells was significantly higher in asymptomatic as compared to symptomatic individuals (Figure 1B). Both CD4$^+$ and CD8$^+$ T cells of symptomatic patients expressed higher levels of activation (CD38$^+$) and exhaustion (PD-1$^+$) markers (Figures 1B and 1C) compared to asymptomatic individuals. Collectively, our study detected increased T cell activation and exhaustion in symptomatic as compared to asymptomatic individuals.

Symptomatic individuals exhibit higher proportions of atypical and memory B cells as compared to asymptomatic individuals

To determine the relationship between clinical presentation and B cell populations, peripheral B cell proportions were compared

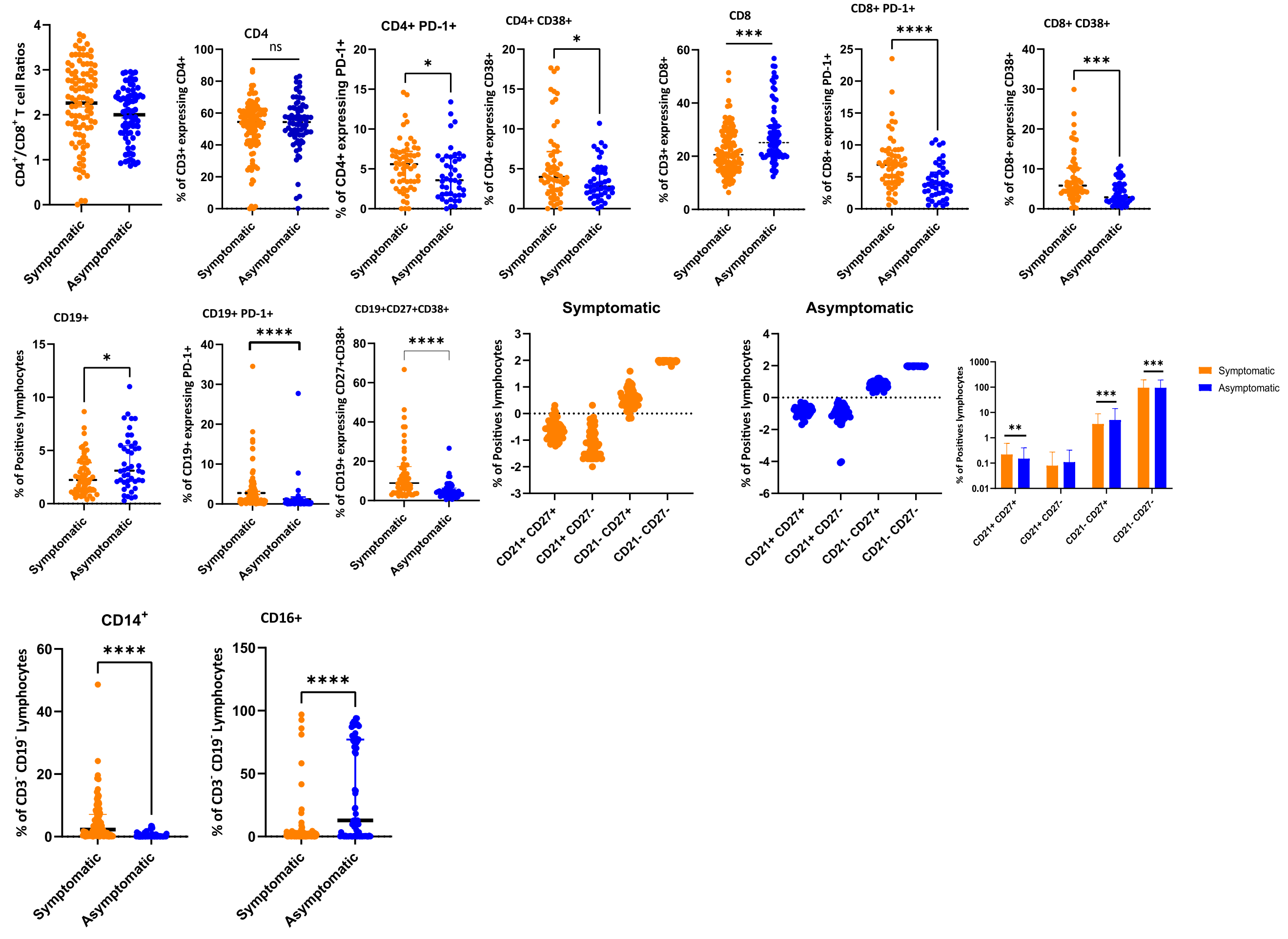

Figure 1. Comparisons of percentage cell proportions in asymptomatic and symptomatic individuals

Shown are CD4+/CD8+ ratios, CD4+ T cells, CD8+ T cells, B cells (CD19+), and antibody-secreting plasma cells (CD27+CD38+). The scatterplots and bar graphs show the proportion of classical memory B cells (CD21+CD27+), naive B cells (CD21+CD27−), atypical memory B cells (CD21−CD27−), activated memory B cells (CD21−CD27+), classical monocytes (CD14+), and non-classical monocytes (CD16+) between symptomatic and asymptomatic individuals. Cell proportions were determined from PBMCs of patients with symptomatic (n = 155) and asymptomatic (n = 62) COVID-19. A horizontal line across the scatterplots shows the median baseline samples per participant, while the lower and upper dotted lines represent the first and third percentiles, respectively. The bar plots show the mean with standard error bars. Statistical significance between symptomatic and asymptomatic patients was determined by a two-tailed Mann-Whitney U test. Multiple comparisons between different groups were tested using Kruskal-Wallis with Dunn's post hoc test (non-significant [ns], $p > 0.05$; *$p < 0.05$; **$p < 0.01$; ****$p < 0.0001$).

between symptomatic and asymptomatic individuals. Although B cell (CD19+) proportions were observed to be higher overall in asymptomatic individuals, symptomatic individuals had a higher proportion of B cells expressing markers of exhaustion (CD19+PD1+) and a higher proportion of antibody-secreting plasma cells (CD19+CD27+CD38+) (Figure 1D). Furthermore, symptomatic individuals had higher proportions of both atypical (CD19+CD21−CD27−) and classical memory B cells (CD19+ CD21−CD27+) when compared to asymptomatic individuals. Activated memory B cells were higher in asymptomatic as compared to symptomatic individuals (Figures 1E and S4). The naive B cell subset (CD19+CD21+CD27+) was not significantly different between symptomatic and asymptomatic individuals (Figures 1E and S4), and the relative abundance of this subset was observed to decrease with time throughout the infection (Figure S3).

We also detected significantly higher proportions of non-classical monocytes (CD16+) in asymptomatic individuals, while classical monocytes (CD14+) were significantly higher in symptomatic individuals (Figure 1F).

Taken together, we demonstrate that symptomatic individuals are characterized by expanded populations of both atypical and memory B cells, while asymptomatic patients displayed more activated B cell phenotypes than symptomatic individuals.

SARS-CoV-2-specific CD4+ and CD8+ T cells are expanded in the periphery of symptomatic patients compared to asymptomatic individuals

To determine whether the SARS-CoV-2-specific immune response differed according to disease status, antigen-specific cytokine production was quantified from PBMCs stimulated

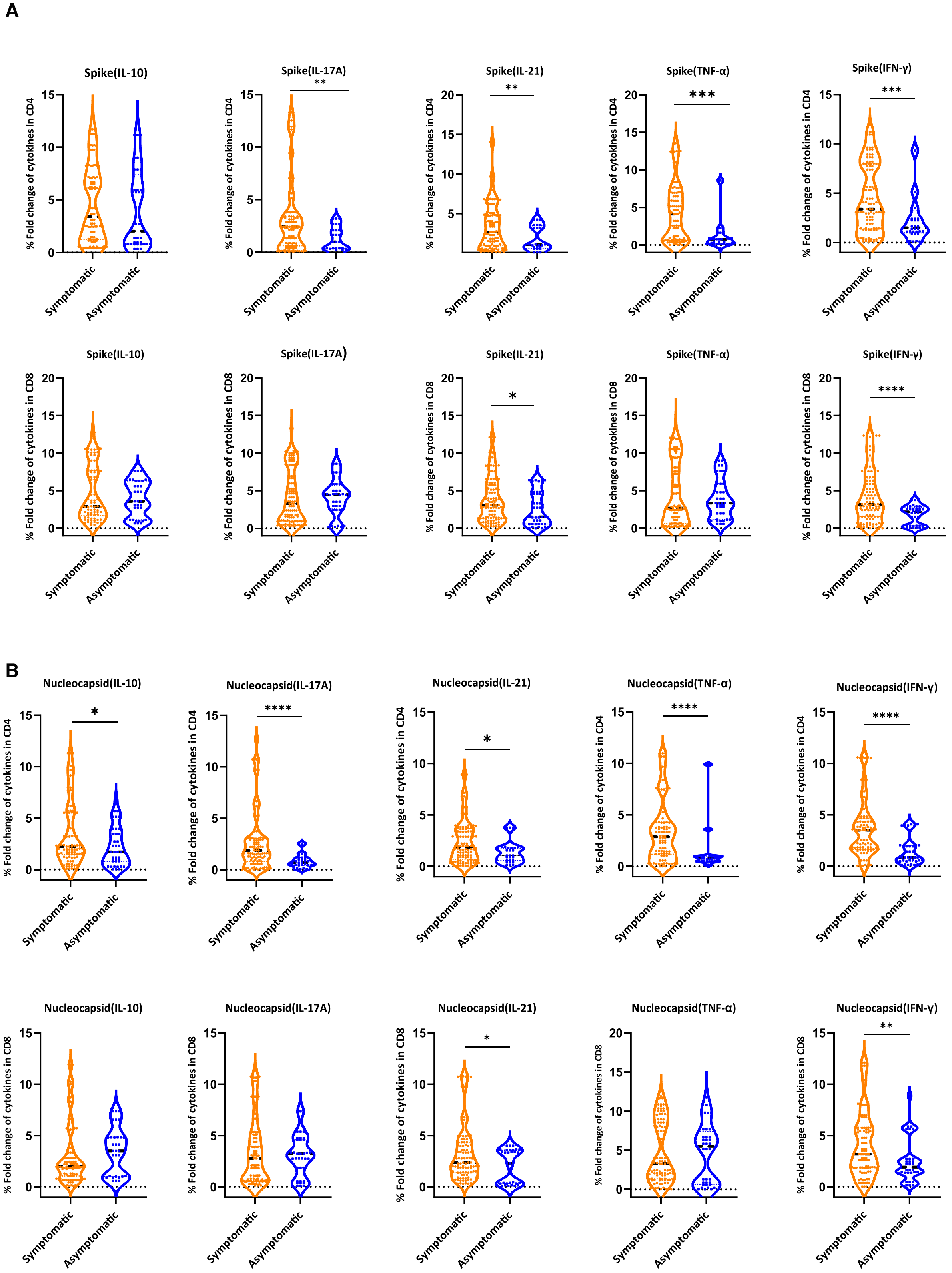

(legend on next page)

with Nucleocapsid and Spike antigens. The pattern of responses produced when cells were stimulated with Spike antigen was generally similar to when the cells were stimulated with the Nucleocapsid antigen (Figure 2). We demonstrated a significantly higher percentage of Spike-specific interferon γ (IFN-γ)-, IL-17A-, and IL-21-producing CD4$^+$ T cells and Spike-specific IFN-γ-, IL-17A-, IL-21-, tumor necrosis factor alpha (TNF-α)-, and IL-10-producing CD8$^+$ T cells in symptomatic compared to asymptomatic individuals. The Nucleocapsid-specific immune response was characterized by increased proportions of IL-21 producing CD4$^+$ T cells and IFN-γ-, IL-17A-, IL-21-, TNF-α-, and IL-10-producing CD8$^+$ T cells in symptomatic as compared to asymptomatic individuals (Figure 2).

In summary, CD8$^+$ and CD4$^+$ T cells were more responsive to re-stimulation with both SARS-CoV-2 antigens in symptomatic compared to the asymptomatic individuals (Figures 1B and 1C).

Patients with asymptomatic COVID-19 are more likely to have elevated malaria antibody responses and reduced production of cytokines than symptomatic patients

To investigate the impact of recent malaria exposure on COVID-19 clinical presentation, we first identified any measurable differences in antibody levels against the most common malarial pathogen in West Africa, *P. falciparum.* Plasma samples from study participants were profiled for antibody responses against selected *P. falciparum* surface antigens: *P. falciparum* circumsporozoite protein (PfCSP), PF3D7_1410700 (E-14), PF3D7_0507400 (E-17), *P. falciparum* merozoites-associated armadillo repeats protein (PfMAAP), and reticulocyte binding-like protein homolog 5 (RH5), indicative of previous *Plasmodium* infection. Of the five antigens used, four (PfCSP, E-14, E-17, and RH5) showed significantly elevated antibody responses in asymptomatic individuals compared with symptomatic individuals (Figures 3A and S5).

A correlation matrix was conducted to examine the association between anti-malaria antibodies and cytokine levels. The results showed that antibodies produced by most antigens (PfCSP, E-14, E-17, and RH5) were negatively correlated with most of the cytokine levels, including the proinflammatory ones (Figure S6).

Taken together, malaria seropositivity appeared to be associated with asymptomatic COVID-19 infection. These data suggested a possible link between *Plasmodium* exposure and COVID-19 severity.

Asymptomatic individuals elicit a higher proportion of polyfunctional T cells against SARS-CoV-2 and *P. falciparum*

To probe further, we examined the production of cytokines from T cells upon PBMC stimulation with *P. falciparum* merozoites lysates. It was observed that *P. falciparum*-specific IL-17A-, IL-21-, TNF-α-, and IL-10-producing CD4$^+$ T cells and *P. falciparum*-specific IFN-γ-, IL-17A-, IL-21-, TNF-α-, and IL-10-producing CD8$^+$ T cells are expressed at a higher level in symptomatic as compared to asymptomatic COVID-19 infection (Figure 4A).

We then determined the ability of patients with COVID-19 disease to elicit polyfunctional T cells upon antigen re-stimulation using the Boolean gating approach. In both CD4$^+$ and CD8$^+$ T cells, significantly higher percentages of triple functionality (IFN-γ/TNF-α/IL-10) and double functionality (IFN-γ/TNF-α) were observed in symptomatic as compared to asymptomatic individuals (Figure 4B). Although the majority of Nucleocapsid-specific CD4$^+$ and CD8$^+$ T cells were mono-functional, the CD4$^+$ T cells appeared to be more polyfunctional than CD8$^+$ T cells. For Spike and *P. falciparum* re-stimulation, both CD4$^+$ and CD8$^+$ T cells showed evidence of polyfunctionality (Figure 4B). In this experiment, symptomatic individuals appeared to produce more polyfunctional specific T cells than asymptomatic individuals (Figure 4B). However, asymptomatic patients appeared to produce a higher overall proportion of polyfunctional T cells as compared to symptomatic patients (Figure 4B). A higher proportion of polyfunctional Nucleocapsid-specific CD4$^+$ and CD8$^+$ T cells was observed in asymptomatic than symptomatic individuals. Moreover, Spike and *P. falciparum*-specific-CD8$^+$ T cells also showed evidence of polyfunctionality in asymptomatic compared to symptomatic patients.

In overall, asymptomatic patients showed a lower specific T cell response to SARS-CoV-2 but a higher overall proportion of polyfunctional T cells (Figure 4B).

DISCUSSION

Most West African countries, including Ghana, have had significantly fewer COVID-19 deaths and high asymptomatic spread compared to other regions.[6–8,32] One of the possible contributing factors is the lower incidence of severe inflammation among patients with COVID-19, as described in our previous study.[33] However, the specific cellular immune mechanisms responsible for these mild phenotypes have not been explored. It had been suggested that immunoregulation induced by previous exposure to infections such as *P. falciparum* could indirectly be protective against severe SARS-CoV-2-mediated disease. Considering that Ghana is a malaria-endemic country in West Africa[34,35] and the centrality of host immunity in COVID-19 prognosis,[36] the present study sought to explore the relationship between previous exposure to malaria and immune responses in SARS-CoV-2-infected individuals. The study further phenotyped and determined the functionality of patient-derived CD4$^+$ and CD8$^+$ T cells from SARS-CoV-2-positive Ghanaians who were either symptomatic or asymptomatic.

Several studies have demonstrated that repeated exposure to *Plasmodium* infection results in an immunological imprint in the host immune system so that inflammation causing disease

Figure 2. Comparison of SARS-CoV-2 antigen-induced cytokines from PBMCs of different study subsets

Shown are the percentage fold change of cytokines by Spike (A) and Nucleocapsid (B) between patients with symptomatic (n = 116) and asymptomatic (n = 44) COVID-19 and negative (n = 74) individuals. The percentage fold changes were calculated by subtracting the percentages of stimulated from unstimulated cells and then dividing by the unstimulated. A horizontal line across the violin plots shows the median cell percentage fold change, while the lower and upper dotted lines represent the 25th and 75th percentiles, respectively. Statistical significance was determined by Mann-Whitney U test (ns, p > 0.05; *p < 0.05; **p < 0.01; ****p < 0.0001).

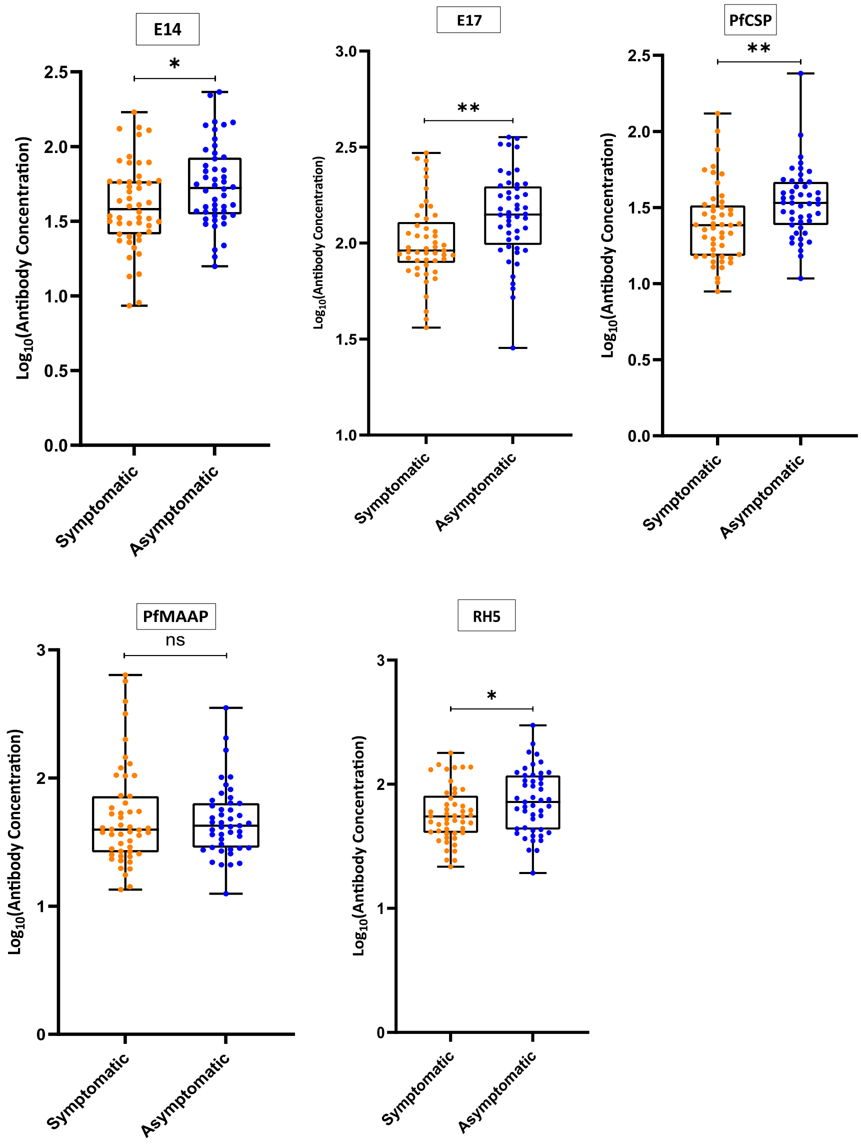

Figure 3. Comparisons of *P. falciparum* exposure in patients with symptomatic and asymptomatic COVID-19

Shown are the immunoglobulin G (IgG) antibody concentrations of patients with symptomatic (n = 50) and asymptomatic (n = 50) COVID-19. The antibodies were tested against *P. falciparum* antigens: PfCSP, E-14, E-17, RH5, and PfMAAP. The median antibody concentration is shown by a horizontal line across the box-and-whisker plots, while the lower and upper dotted lines represent the first and third quartiles, respectively. Statistical significance was determined by two-tailed Mann-Whitney U test (ns, $p > 0.05$; *$p < 0.05$; **$p < 0.01$; ****$p < 0.0001$).

pathogenesis is downregulated.[16] Thus, with multiple infections, the host's immune system is suppressed, allowing the host to avoid clinical disease while remaining asymptomatic. Certainly, host immune modification caused by one pathogen can impact the responses generated against a different or co-infecting pathogen.[37]

A study by Niraj et al. found that individuals co-infected with malaria and COVID-19 recovered faster from COVID-19 than those singly infected with COVID-19.[38] This finding is in line with the current study, where we found higher *P. falciparum* antibody titers in patients with asymptomatic COVID-19 than in symptomatic patients. In addition, individuals with decreased inflammatory responses to *P. falciparum* antigens were mostly patients with asymptomatic COVID-19.

Although there are no experimental studies exploring the relationship between malaria and mild COVID-19, a study in Uganda observed a link between previous higher exposure to malaria and COVID-19 symptoms.[23] This seemed to suggest that previous exposure to the parasite was protective against symptomatic COVID-19. It has also been reported that high *P. falciparum*-specific antibody titers and breadth are protective against severe diseases.[39] Conversely, symptomatic individuals exhibited higher T cell responses against *Plasmodium* antigens than asymptomatic individuals.

Taken together, these data support a protective role of *P. falciparum* infection against severe COVID-19. This may also be the reason why Ghana's population and much of West Africa have reported disproportionately higher percentages of asymptomatic SARS-CoV-2 infections compared to other regions.[32,40]

In immunophenotyping, the proportion of $CD4^+$ T cells in peripheral blood was comparable between symptomatic and asymptomatic individuals. However, the T cells from the symptomatic patients demonstrated increased activation and exhaustion compared to asymptomatic individuals, consistent with our previous findings.[33] The increased activation and exhaustion of immune cells suggested a diminished immune response and higher cytokine production in symptomatic patients, as reported previously.[41,42] Thus, increased expression of T cell activation markers with concurrent production of cytokines in the symptomatic individuals seen here is consistent with the role of cytokines in COVID-19 pathogenesis.

B cells from symptomatic individuals expressed higher levels of exhaustion markers and increased proportions of atypical and classic memory B cells compared to asymptomatic individuals. Atypical memory B cells are antigen-experienced B cells, characterized as dysfunctional or impaired B cells, and are usually associated with chronic infection, including malaria.[43,44] Classical memory B cells are matured cells characterized by their ability to differentiate into antibody-secreting plasma cells and can persist long term.[44]

CellPress
OPEN ACCESS

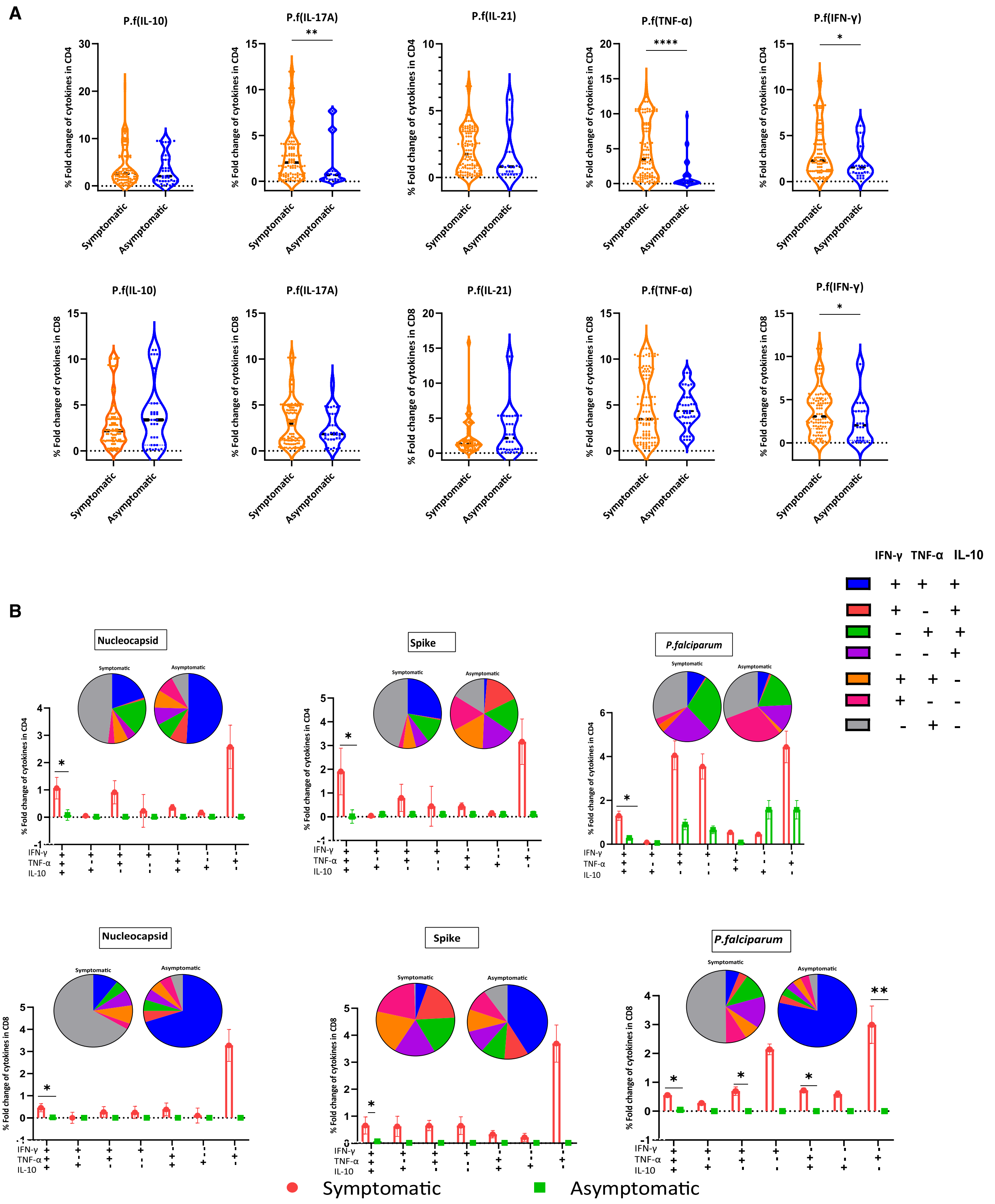

(legend on next page)

This study further showed increased antibody-secreting plasma cells in symptomatic compared to asymptomatic patients. Although there are limited data comparing the percentage of cell proportions of memory B cells in patients with symptomatic and asymptomatic COVID-19, an expansion of atypical memory B cells and antibody-secreting plasma cells has generally been found in patients with severe COVID-19.[45,46] This was mostly detected in patients who died rather than survivors. Conversely, the classical memory B cells declined in patients with severe COVID-19.[45] However, our study showed a higher frequency of these classical memory B cells in symptomatic patients. Furthermore, the current study presented a higher percentage of activated memory B cells in asymptomatic than in symptomatic patients.

Classical monocytes, associated with the initiation of pro-inflammatory cytokine responses,[47] were more elevated in symptomatic compared to asymptomatic individuals, providing a possible explanation for the increased cytokines in symptomatic individuals.[33] The study findings are in line with studies from other regions where higher activation and exhaustion of cells such as CD8$^+$ T cells in the peripheral blood of severe/symptomatic individual cases was revealed.[30,48,49] The lower frequencies of B cells may suggest that the cells in symptomatic individuals may have migrated to tissues or lymph nodes in response to the virus,[50] while in asymptomatic patients, more cells remain circulating in the bloodstream due to lower immune activation.

The study also explored the functionality of SARS-CoV-2-specific T cells using Nucleocapsid and Spike proteins. Ghanaian SARS-CoV-2 patients displayed similar specific responses from Nucleocapsid and Spike in both CD4$^+$ and CD8$^+$ T cells. The similarity between Nucleocapsid and Spike immune responses in this cohort was also shown by our previous study, where anti-Nucleocapsid and anti-Spike antibodies were comparable.[33] However, a higher percentages of anti-Spike antibodies than anti-Nucleocapsid antibodies[51,52] have been linked to enhanced disease severity.

When we assessed functional responses to the SARS-CoV-2 antigens (Spike and Nucleocapsid), T cell responses seemed to be more strongly elicited in symptomatic compared to asymptomatic individuals. This corroborates the increased activation status of T cells in symptomatic individuals observed in this study, indicating that the immune system is more activated in symptomatic as compared to asymptomatic patients.

The present study also detected a higher percentage of SARS-CoV-2 antigen-specific T cells that simultaneously produce IL-10 and key inflammatory cytokines (IFN-γ and TNF-α) in symptomatic than asymptomatic patients. A higher antigen load in symptomatic participants likely resulted in a higher percentage of polyfunctional T cell responses. T cells tend to be more activated and produce increased cytokines following stronger antigen stimulation.[53]

In terms of proportions, asymptomatic patients appeared to produce more polyfunctional T cells as compared to symptomatic patients, suggesting a potential protection of this particular group. Polyfunctional T cells, particularly CD8$^+$ T cells, are known to have a more functional immune response, thus providing more effective immune responses to pathogens.[54] Polyfunctional T cells have also been associated with protection against viral infections due to their potent effector capability, and studies have shown their presence among convalescent patients with mild COVID-19.[55–57]

Polyfunctional T cells possess potent effector capabilities and have been linked to shielding against viral infections. Though no comparative studies have been conducted previously regarding the polyfunctionality of T cells in symptomatic and asymptomatic patients, polyfunctional T cells have been observed among convalescent patients with mild COVID-19.[55–57]

In conclusion, this study builds on our initial report, which reported low levels of proinflammatory cytokines in the peripheral blood of Ghanaian patients with COVID-19, particularly in asymptomatic individuals. While more studies are needed, we show here that *Plasmodium* infection and exposure may play a role in the immune response to SARS-CoV-2 infection and may be linked to the low severity of COVID-19 in Ghana and West Africa.

Limitations of the study

We acknowledge that the sample size for the analysis of the associations between malaria exposure and pathology of SARS-CoV-2 infection was small. Additionally, there were some missing metadata for a few of the participants. Due to the limited sample size, we also grouped mild and severe cases together as "symptomatic" as opposed to dividing them and comparing them with asymptomatic patients, as has been done by other published studies. It was unnecessary to stratify by vaccination status, since none of the asymptomatic individuals were vaccinated. Despite these limitations, our findings do suggest a possible link between previous malaria exposure and reduced COVID-19 severity in malaria-endemic regions. Further mechanistic investigations will be required to definitively establish the key mediators of this phenomenon.

STAR★METHODS

Detailed methods are provided in the online version of this paper and include the following:

- KEY RESOURCES TABLE

Figure 4. Comparisons of the percentage fold change of cytokines by *P. falciparum* and polyfunctional T cells between patients with symptomatic and asymptomatic COVID-19

(A) Comparison of percentage fold changes of cytokines by *P. falciparum* between patients with symptomatic (n = 116) and asymptomatic (n = 44) COVID-19 disease. The percentage fold changes were calculated by subtracting the unstimulated from stimulated cells and dividing by the unstimulated. A horizontal line across the violin plots shows the median percentage fold change, while the lower and upper dotted lines represent the 25th and 75th percentiles, respectively. Statistical significance was determined by a two-tailed Mann-Whitney U test (ns, $p > 0.05$; *$p < 0.05$; **$p < 0.01$; ****$p < 0.0001$).

(B) Percentage comparisons of polyfunctional T cells in patients with symptomatic (n = 116) and asymptomatic (n = 44) COVID-19.

The interleaved pie and bar charts represent the percentage of CD4$^+$ (A) and CD8$^+$ (B), simultaneously producing single, double, and triple cytokines after *in vitro* stimulation with Nucleocapsid, Spike, and *P. falciparum* lysate. The pie charts represent the pattern of cytokine production in symptomatic and asymptomatic individuals. Data are expressed as means ± standard error of the mean. Statistical significance between symptomatic and asymptomatic patients were determined by a two-tailed Mann-Whitney U test (ns, $p > 0.05$; *$p < 0.05$).

CellPress
OPEN ACCESS

- RESOURCE AVAILABILITY
  - Lead contact
  - Materials availability
  - Data and code availability
- EXPERIMENTAL MODEL AND STUDY PARTICIPANT DETAILS
- METHOD DETAILS
  - Ethics approval and consent to participate
  - Sample collection and preparation
  - PBMC isolation, cryopreservation, and thawing
  - Culture of malaria parasites and isolation of merozoites
  - Immunophenotyping
  - *In vitro* stimulation and intracellular cytokine staining
  - Detection of anti-*Plasmodium falciparum* antibodies
- QUANTIFICATION AND STATISTICAL ANALYSIS

SUPPLEMENTAL INFORMATION

Supplemental information can be found online at https://doi.org/10.1016/j.celrep.2024.114533.

ACKNOWLEDGMENTS

With their confirmed permission, we sincerely thank all study participants for their contributions. We are also grateful to all clinical staff for their support at participating hospitals. We are thankful to Dr. Christèle N. Tchopba for critical review of the manuscript. This publication is based on research funded in part by the Bill & Melinda Gates Foundation (INV-036307); in part by grants from The Rockefeller Foundation (2021 HTH 006); in part by the Cambridge-Africa Alborada Research Fund-COVID-19 Emergency Award (G109329); in part by the Crick African Network (CAN/A00004/1 and CAN/A00003/1), which receives its funding from the UK's Global Challenges Research Fund (MR/P028071/1); and by the Francis Crick Institute, which receives its core funding from Cancer Research UK (FC1001647), the UK Medical Research Council (FC1001647), and the Wellcome Trust (FC1001647). K.T. is supported by a WACCBIP-World Bank ACE PhD fellowship (WACCBIP+NCDs: Awandare) and a WANIDA (West African Network of Infectious Diseases ACEs) doctoral scholarship (WAN100634R). This scholarship package is funded by the AFD, IRD, and World Bank under the African Centers of Excellence (ACE) Partner Project. For open access, the author has applied a CC BY public copyright license to any author-accepted manuscript version arising from this submission. The views expressed in this publication are those of the authors and not necessarily those of the funders.

AUTHOR CONTRIBUTIONS

Conceptualization ideas, G.A.A., P.K.Q., Y.B., and D.A.P.; methodology, K.T., F.N., D.A.P., N.N., P.K.Q., Y.B., and Y.A.; validation, K.T., D.A.P., F.N., and K.S.; formal analysis, K.T., D.A.P., F.N., and K.S.; investigation, K.T., B.T., D.A.P., Y.B., Y.A., P.K.Q., F.N., D.D., A.C., K.O.A., D.Q., C.A.A., F.A., S.A., I.A.O., C.K., G.A., E.A., W.K.A., and J.K.M.; resources, G.A.A., P.K.Q., Y.B., and Y.A.; writing – original draft, K.T., D.A.P., and P.K.Q.; writing – review and editing, P.K.Q., Y.B., K.S., and G.A.A.; visualization, K.T., D.A.P., P.K.Q., and Y.B.; supervision, P.K.Q. and Y.B.; project administration, K.T. and B.T.; funding acquisition, P.K.Q., Y.B., and G.A.A. All authors read and approved the final version.

DECLARATION OF INTERESTS

The authors declare no competing interests.

Received: August 31, 2023
Revised: October 17, 2023
Accepted: July 9, 2024
Published: July 24, 2024

REFERENCES

1. (2020). Worldometers. Coronavirus cases. Worldometers.info. https://doi.org/10.26439/iusetpraxis2020.n50-51.5049.
2. Zeng, H., Ma, Y., Zhou, Z., Liu, W., Huang, P., Jiang, M., Liu, Q., Chen, P., Luo, H., and Chen, Y. (2021). Spectrum and Clinical Characteristics of Symptomatic and Asymptomatic Coronavirus Disease 2019 (COVID-19) With and Without Pneumonia. Front. Med. *8*, 645651–645659. https://doi.org/10.3389/fmed.2021.645651.
3. Osei, S.A., Biney, R.P., Anning, A.S., Nortey, L.N., and Ghartey-Kwansah, G. (2022). Low incidence of COVID-19 case severity and mortality in Africa; Could malaria co-infection provide the missing link? BMC Infect. Dis. *22*, 78. https://doi.org/10.1186/s12879-022-07064-4.
4. Morang, C.M., Ngoi, J.M., Gyam, J., Amuzu, D.S.Y., Nuertey, B.D., Soglo, P.M., Appiah, V., Asante, I.A., Owusu-Oduro, P., Armoo, S., et al. (2022). Genetic diversity of SARS-CoV-2 infections in Ghana from 2020-2021. Nat. Commun. *13*, 1–11. https://doi.org/10.1038/s41467-022-30219-5.
5. Wilkinson, E., Giovanetti, M., Tegally, H., San, J.E., Lessells, R., Cuadros, D., Martin, D.P., Rasmussen, D.A., Zekri, A.R.N., Sangare, A.K., et al. (2021). A year of genomic surveillance reveals how the SARS-CoV-2 pandemic unfolded in Africa. Science (80-) *374*, 423–431. https://doi.org/10.1126/science.abj4336.
6. Forku, R. (2021). WHO praises Africa ' s response as virus cases decline. Health,Africa. https://www.aa.com.tr/en/africa/who-praises-africas-response-as-virus-cases-decline/1984469.
7. Quashie, P.K., Mutungi, J.K., Dzabeng, F., Oduro-Mensah, D., Opurum, P.C., Tapela, K., Udoakang, A.J., Asante, I., Paemka, L., Kumi-Ansah, F., et al. (2021). Trends of severe acute respiratory syndrome coronavirus 2 (SARS-CoV-2) antibody prevalence in selected regions across Ghana. Wellcome Open Res. *6*, 173. https://doi.org/10.12688/wellcomeopenres.16890.1.
8. Uyoga, S., Adetifa, I.M.O., Karanja, H.K., Nyagwange, J., Tuju, J., Wanjiku, P., Aman, R., Mwangangi, M., Amoth, P., Kasera, K., et al. (2021). Seroprevalence of anti-SARS-CoV-2 IgG antibodies in Kenyan blood donors. Science (80-) *371*, 79–82. https://doi.org/10.1126/science.abe1916.
9. Lawal, Y. (2021). Africa's low COVID-19 mortality rate: A paradox? Int. J. Infect. Dis. *102*, 118–122. https://doi.org/10.1016/j.ijid.2020.10.038.
10. Oleribe, O.O., Suliman, A.A.A., Taylor-Robinson, S.D., and Corrah, T. (2021). Possible reasons why sub-saharan africa experienced a less severe COVID-19 pandemic in 2020. J. Multidiscip. Healthc. *14*, 3267–3271. https://doi.org/10.2147/JMDH.S331847.
11. Kalungi, A., Kinyanda, E., Akena, D.H., Kaleebu, P., and Bisangwa, I.M. (2021). Less Severe Cases of COVID-19 in Sub-Saharan Africa: Could Co-infection or a Recent History of Plasmodium falciparum Infection Be Protective? Front. Immunol. *12*, 565625. https://doi.org/10.3389/fimmu.2021.565625.
12. WHO (2022). Malaria (World health Organisation). https://www.who.int/health-topics/diabetes#tab=tab_1.
13. Ajayi, I.O.O., Ajumobi, O.O., and Falade, C. (2020). Malaria and COVID-19: commonalities, intersections and implications for sustaining malaria control. Pan Afr. Med. J. *37*, 1–10. https://doi.org/10.11604/pamj.supp.2020.37.1.25738.
14. Hussein, M.I.H., Albashir, A.A.D., Elawad, O.A.M.A., and Homeida, A. (2020). Malaria and COVID-19: unmasking their ties. Malar. J. *19*, 457–510. https://doi.org/10.1186/s12936-020-03541-w.
15. Doolan, D.L., Dobaño, C., and Baird, J.K. (2009). Acquired immunity to Malaria. Clin. Microbiol. Rev. *22*, 13–36. https://doi.org/10.1128/CMR.00025-08.
16. Bediako, Y., Adams, R., Reid, A.J., Valletta, J.J., Ndungu, F.M., Sodenkamp, J., Mwacharo, J., Ngoi, J.M., Kimani, D., Kai, O., et al. (2019). Repeated clinical malaria episodes are associated with modification of the immune system in children. BMC Med. *17*, 60. https://doi.org/10.1186/s12916-019-1292-y.

17. Ademolue, T.W., Aniweh, Y., Kusi, K.A., and Awandare, G.A. (2017). Patterns of inflammatory responses and parasite tolerance vary with malaria transmission intensity. Malar. J. *16*, 145–213. https://doi.org/10.1186/s12936-017-1796-x.

18. Guha, R., Mathioudaki, A., Doumbo, S., Doumtabe, D., Skinner, J., Arora, G., Siddiqui, S., Li, S., Kayentao, K., Ongoiba, A., et al. (2021). Plasmodium falciparum malaria drives epigenetic reprogramming of human monocytes toward a regulatory phenotype. PLoS Pathog. *17*, 10094300–e1009522. https://doi.org/10.1371/journal.ppat.1009430.

19. Keller, C.C., Yamo, O., Ouma, C., Ong'echa, J.M., Ounah, D., Hittner, J.B., Vulule, J.M., and Perkins, D.J. (2006). Acquisition of hemozoin by monocytes down-regulates interleukin-12 p40 (IL-12p40) transcripts and circulating IL-12p70 through an IL-10-dependent mechanism: In vivo and in vitro findings in severe malarial anemia. Infect. Immun. *74*, 5249–5260. https://doi.org/10.1128/IAI.00843-06.

20. Oduro-Mensah, D., Oduro-Mensah, E., Quashie, P., Awandare, G., and Okine, L. (2021). Explaining the unexpected COVID-19 trends and potential impact across Africa. F1000Res. *10*, 1177–1214. https://doi.org/10.12688/f1000research.74363.2.

21. Tsoka-Gwegweni, J.M. (2022). Status of malaria and its implications for elimination in an endemic province of South Africa: retrospective analysis. Pan Afr. Med. J. *41*, 275–278. https://doi.org/10.11604/pamj.2022.41.275.26774.

22. Bwire, G., Ario, A.R., Eyu, P., Ocom, F., Wamala, J.F., Kusi, K.A., Ndeketa, L., Jambo, K.C., Wanyenze, R.K., and Talisuna, A.O. (2022). The COVID-19 pandemic in the African continent. BMC Med. *20*, 167–223. https://doi.org/10.1186/s12916-022-02367-4.

23. Achan, J., Serwanga, A., Wanzira, H., Kyagulanyi, T., Nuwa, A., Magumba, G., Kusasira, S., Sewanyana, I., Tetteh, K., Drakeley, C., et al. (2022). Current malaria infection, previous malaria exposure, and clinical profiles and outcomes of COVID-19 in a setting of high malaria transmission: an exploratory cohort study in Uganda. Lancet. Microbe *3*, e62–e71. https://doi.org/10.1016/S2666-5247(21)00240-8.

24. Woodford, J., Sagara, I., Diawara, H., Assadou, M.H., Katile, A., Attaher, O., Issiaka, D., Santara, G., Soumbounou, I.H., Traore, S., et al. (2022). Recent malaria does not substantially impact COVID-19 antibody response or rates of symptomatic illness in communities with high malaria and COVID-19 transmission in Mali, West Africa. Front. Immunol. *13*, 959697–959698. https://doi.org/10.3389/fimmu.2022.959697.

25. Chen, G., Wu, D., Guo, W., Cao, Y., Huang, D., Wang, H., Wang, T., Zhang, X., Chen, H., Yu, H., et al. (2020). Clinical and immunological features of severe and moderate coronavirus disease 2019. J. Clin. Invest. *130*, 2620–2629. https://doi.org/10.1172/JCI137244.

26. Wang, D., Hu, B., Hu, C., Zhu, F., Liu, X., Zhang, J., Wang, B., Xiang, H., Cheng, Z., Xiong, Y., et al. (2020). Clinical Characteristics of 138 Hospitalized Patients with 2019 Novel Coronavirus-Infected Pneumonia in Wuhan, China. JAMA, J. Am. Med. Assoc. *323*, 1061–1069. https://doi.org/10.1001/jama.2020.1585.

27. Thevarajan, I., Nguyen, T.H.O., Koutsakos, M., Druce, J., Caly, L., van de Sandt, C.E., Jia, X., Nicholson, S., Catton, M., Cowie, B., et al. (2020). Breadth of concomitant immune responses prior to patient recovery: a case report of non-severe COVID-19. Nat. Med. *26*, 453–455. https://doi.org/10.1038/s41591-020-0820-9.

28. Liao, M., Liu, Y., Yuan, J., Wen, Y., Xu, G., Zhao, J., Cheng, L., Li, J., Wang, X., Wang, F., et al. (2020). Single-cell landscape of bronchoalveolar immune cells in patients with COVID-19. Nat. Med. *26*, 842–844. https://doi.org/10.1038/s41591-020-0901-9.

29. Carsetti, R., Zaffina, S., Piano Mortari, E., Terreri, S., Corrente, F., Capponi, C., Palomba, P., Mirabella, M., Cascioli, S., Palange, P., et al. (2020). Different Innate and Adaptive Immune Responses to SARS-CoV-2 Infection of Asymptomatic, Mild, and Severe Cases. Front. Immunol. *11*, 610300–610316. https://doi.org/10.3389/fimmu.2020.610300.

30. Li, M., Zhang, Y., Lu, J., Li, L., Gao, H., Ma, C., Dai, E., and Wei, L. (2021). Asymptomatic covid-19 individuals tend to establish relatively balanced innate and adaptive immune responses. Pathogens *10*, 1105. https://doi.org/10.3390/pathogens10091105.

31. Le Bert, N., Clapham, H.E., Tan, A.T., Chia, W.N., Tham, C.Y.L., Lim, J.M., Kunasegaran, K., Tan, L.W.L., Dutertre, C.A., Shankar, N., et al. (2021). Highly functional virus-specific cellular immune response in asymptomatic SARS-CoV-2 infection. J. Exp. Med. *218*, e20202617. https://doi.org/10.1084/JEM.20202617.

32. Olayanju, O., Bamidele, O., Edem, F., Eseile, B., Amoo, A., Nwaokenye, J., Udeh, C., Oluwole, G., Odok, G., and Awah, N. (2021). SARS-CoV-2 seropositivity in asymptomatic frontline health workers in Ibadan, Nigeria. Am. J. Trop. Med. Hyg. *104*, 91–94. https://doi.org/10.4269/AJTMH.20-1235.

33. Tapela, K., Oyawoye, F.O., Olwal, C.O., Opurum, P.C., Amponsah, J.A., Segbedzi, K.A.L., Tetteh, B., Kumi-Ansah, F., Mutungi, J.K., Obodai, E., et al. (2022). Probing SARS-CoV-2-positive plasma to identify potential factors correlating with mild COVID-19 in Ghana, West Africa. BMC Med. *20*, 370. https://doi.org/10.1186/s12916-022-02571-2.

34. WHO (2021). Private Sector Support Key in Achieving Zero Malaria in Ghana | WHO | Regional Office for Africa (World Health Organization). https://www.afro.who.int/news/private-sector-support-key-achieving-zero-malaria-ghana.

35. Awine, T., Malm, K., Bart-Plange, C., and Silal, S.P. (2017). Towards malaria control and elimination in Ghana: Challenges and decision making tools to guide planning. Glob. Health Action *10*, 1381471–1381479. https://doi.org/10.1080/16549716.2017.1381471.

36. Velavan, T.P., Pallerla, S.R., Rüter, J., Augustin, Y., Kremsner, P.G., Krishna, S., and Meyer, C.G. (2021). Host genetic factors determining COVID-19 susceptibility and severity. EBioMedicine *72*, 103629. https://doi.org/10.1016/j.ebiom.2021.103629.

37. Okon, E.M., Okocha, R.C., Taiwo, A.B., Michael, F.B., and Bolanle, A.M. (2023). Dynamics of co-infection in fish: A review of pathogen-host interaction and clinical outcome. Fish Shellfish Immunol. Rep. *4*, 100096. https://doi.org/10.1016/j.fsirep.2023.100096.

38. Mahajan, N.N., Gajbhiye, R.K., Bahirat, S., Lokhande, P.D., Mathe, A., Rathi, S., Warty, N., Mahajan, K.N., Srivastava, V., Kuppusamy, P., and Mohite, S.C. (2021). Co-infection of malaria and early clearance of SARS-CoV-2 in healthcare workers. J. Med. Virol. *93*, 2431–2438. https://doi.org/10.1002/jmv.26760.

39. Osier, F.H.A., Fegan, G., Polley, S.D., Murungi, L., Verra, F., Tetteh, K.K.A., Lowe, B., Mwangi, T., Bull, P.C., Thomas, A.W., et al. (2008). Breadth and magnitude of antibody responses to multiple Plasmodium falciparum merozoite antigens are associated with protection from clinical malaria. Infect. Immun. *76*, 2240–2248. https://doi.org/10.1128/IAI.01585-07.

40. Kenu, E., Odikro, M.A., Malm, K.L., Asiedu-Bekoe, F., Noora, C.L., Frimpong, J.A., Calys-Tagoe, B., and Koram, K.A. (2020). Epidemiology of COVID-19 outbreak in Ghana, 2020. Ghana Med. J. *54*, 5–15. https://doi.org/10.4314/GMJ.V54I4S.3.

41. Ruan, Q., Yang, K., Wang, W., Jiang, L., and Song, J. (2020). Clinical predictors of mortality due to COVID-19 based on an analysis of data of 150 patients from Wuhan, China. Intensive Care Med. *46*, 846–848. https://doi.org/10.1007/s00134-020-05991-x.

42. Queiroz, M.A.F., Neves, P.F.M.D., Lima, S.S., Lopes, J.d.C., Torres, M.K.d.S., Vallinoto, I.M.V.C., Bichara, C.D.A., Dos Santos, E.F., de Brito, M.T.F.M., da Silva, A.L.S., et al. (2022). Cytokine Profiles Associated With Acute COVID-19 and Long COVID-19 Syndrome. Front. Cell. Infect. Microbiol. *12*, 922422–922511. https://doi.org/10.3389/fcimb.2022.922422.

43. Pérez-Mazliah, D., Gardner, P.J., Schweighoffer, E., McLaughlin, S., Hosking, C., Tumwine, I., Davis, R.S., Potocnik, A.J., Tybulewicz, V.L., and Langhorne, J. (2018). Plasmodium-specific atypical memory B cells are short-lived activated B cells. Elife *7*, 1–28. https://doi.org/10.7554/eLife.39800.

44. Good-Jacobson, K.L. (2018). Strength in diversity: Phenotypic, functional, and molecular heterogeneity within the memory B cell repertoire. Immunol. Rev. *284*, 67–78. https://doi.org/10.1111/imr.12663.

CellPress
OPEN ACCESS

45. Oliviero, B., Varchetta, S., Mele, D., Mantovani, S., Cerino, A., Perotti, C.G., Ludovisi, S., and Mondelli, M.U. (2020). Expansion of atypical memory B cells is a prominent feature of COVID-19. Cell. Mol. Immunol. *17*, 1101–1103. https://doi.org/10.1038/s41423-020-00542-2.

46. Wildner, N.H., Ahmadi, P., Schulte, S., Brauneck, F., Kohsar, M., Lütgehetmann, M., Beisel, C., Addo, M.M., Haag, F., and Schulze Zur Wiesch, J. (2021). B cell analysis in SARS-CoV-2 versus malaria: Increased frequencies of plasmablasts and atypical memory B cells in COVID-19. J. Leukoc. Biol. *109*, 77–90. https://doi.org/10.1002/JLB.5COVA0620-370RR.

47. Narasimhan, P.B., Marcovecchio, P., Hamers, A.A.J., and Hedrick, C.C. (2019). Nonclassical Monocytes in Health and Disease. Annu. Rev. Immunol. *37*, 439–456. https://doi.org/10.1146/annurev-immunol-042617-053119.

48. Zhou, R., To, K.K.W., Wong, Y.C., Liu, L., Zhou, B., Li, X., Huang, H., Mo, Y., Luk, T.Y., Lau, T.T.K., et al. (2020). Acute SARS-CoV-2 Infection Impairs Dendritic Cell and T Cell Responses. Immunity *53*, 864–877.e5. https://doi.org/10.1016/j.immuni.2020.07.026.

49. Rupp, J., Dreo, B., Gütl, K., Fessler, J., Moser, A., Haditsch, B., Schilcher, G., Matzkies, L.M., Steinmetz, I., Greinix, H., and Stradner, M.H. (2021). T Cell Phenotyping in Individuals Hospitalized with COVID-19. J. Immunol. *206*, 1478–1482. https://doi.org/10.4049/jimmunol.2001034.

50. Hampton, H.R., and Chtanova, T. (2019). Lymphatic migration of immune cells. Front. Immunol. *10*, 1168–1223. https://doi.org/10.3389/fimmu.2019.01168.

51. Legros, V., Denolly, S., Vogrig, M., Boson, B., Siret, E., Rigaill, J., Pillet, S., Grattard, F., Gonzalo, S., Verhoeven, P., et al. (2021). A longitudinal study of SARS-CoV-2-infected patients reveals a high correlation between neutralizing antibodies and COVID-19 severity. Cell. Mol. Immunol. *18*, 318–327. https://doi.org/10.1038/s41423-020-00588-2.

52. Menon, V., Shariff, M.A., Perez Gutierrez, V., Carreño, J.M., Yu, B., Jawed, M., Gossai, M., Valdez, E., Pillai, A., Venugopal, U., et al. (2021). Longitudinal humoral antibody response to SARS-CoV-2 infection among healthcare workers in a New York City hospital. BMJ Open *11*, 0510455–e51111. https://doi.org/10.1136/bmjopen-2021-051045.

53. Kwon, J.S., Kim, J.Y., Kim, M.C., Park, S.Y., Kim, B.N., Bae, S., Cha, H.H., Jung, J., Kim, M.J., Lee, M.J., et al. (2020). Factors of severity in patients with COVID-19: Cytokine/Chemokine concentrations, viral load, and antibody responses. Am. J. Trop. Med. Hyg. *103*, 2412–2418. https://doi.org/10.4269/ajtmh.20-1110.

54. Foley, J.F. (2012). Immunology: Polyfunctional T cells. Sci. Signal. *5*, 8–10. https://doi.org/10.1126/scisignal.2002929.

55. Zuo, J., Dowell, A.C., Pearce, H., Verma, K., Long, H.M., Begum, J., Aiano, F., Amin-Chowdhury, Z., Hoschler, K., Brooks, T., et al. (2021). Robust SARS-CoV-2-specific T cell immunity is maintained at 6 months following primary infection. Nat. Immunol. *22*, 620–626. https://doi.org/10.1038/s41590-021-00902-8.

56. van der Ploeg, K., Kirosingh, A.S., Mori, D.A.M., Chakraborty, S., Hu, Z., Sievers, B.L., Jacobson, K.B., Bonilla, H., Parsonnet, J., Andrews, J.R., et al. (2022). TNF-α+ CD4+ T cells dominate the SARS-CoV-2 specific T cell response in COVID-19 outpatients and are associated with durable antibodies. Cell Rep. Med. *3*, 100640. https://doi.org/10.1016/j.xcrm.2022.100640.

57. Breton, G., Mendoza, P., Hägglöf, T., Oliveira, T.Y., Schaefer-Babajew, D., Gaebler, C., Turroja, M., Hurley, A., Caskey, M., Nussenzweig, T.C., et al. (2021). Persistent cellular immunity to SARS-CoV-2 infection. J. Exp. Med. *218*, e20202515. https://doi.org/10.1084/JEM.20202515.

STAR★METHODS

KEY RESOURCES TABLE

REAGENT or RESOURCE	SOURCE	IDENTIFIER
**Antibodies**		
CD3-BUV 395	BioLegend, Inc	564001
CD8-APC H7	BioLegend, Inc	641400
CD4-PE	BioLegend, Inc	317410
CD19-BV 785	BioLegend, Inc	302240
CD21-BB 515	BioLegend, Inc	564755
CD27-PE/Cy7	BioLegend, Inc	356412
CD14-APC	BioLegend, Inc	301808
PD-1-BUV 737	BioLegend, Inc	612791
CD38-BV 605	BioLegend, Inc	303532
CD16-PerCP/cy5.5	BioLegend, Inc	360712
HLA-DR-BV 510	BioLegend, Inc	307646
Alexa Fluor 700	BioLegend, Inc	564997
CD49d	BioLegend, Inc	555501
CD28	BioLegend, Inc	302902
IL-10-PE/Cy7	BioLegend, Inc	501420
IL-17A-BV 510	BioLegend, Inc	563295
IL-21 BV-421	BioLegend, Inc	564755
TNF-α-FITC	BioLegend, Inc	502906
IFN-γ-BV 786	BioLegend, Inc	502542
**Chemicals, peptides, and recombinant proteins**		
SARS-CoV-2 Spike	Ab 273063	Abcam
Nucleocapsid	Ab 273530	Abcam
**Experimental models: Organisms/strains**		
P. falciparum: Strain *(Dd2)*		N/A
**Software and algorithms**		
FlowJo	BD Life Sciences	v10.8

RESOURCE AVAILABILITY

Lead contact

Further information and requests for resources and reagents should be directed to and will be fulfilled by the lead contact, Yaw Bediako (ybediako@ug.edu.gh).

Materials availability

This study did not generate new unique reagents.

Data and code availability

- Data reported in this paper will be shared by the lead contact upon request.
- This paper does not report original code
- Any additional information required to reanalyze the data reported in this work paper is available from the lead contact upon request.

EXPERIMENTAL MODEL AND STUDY PARTICIPANT DETAILS

Participants were recruited among the Ghanaian population during the ongoing COVID-19 pandemic in a mixed-methods study consisting of a baseline snapshot analysis from $n = 217$ patients and longitudinal analysis for a subset of patients ($n = 82$). All participants

were above the age of 4 years, females not pregnant, and had received at least one positive COVID-19 PCR test immediately before baseline sample collection. Samples were collected from The Greater Accra Regional Hospital in Accra and Cape Coast Teaching Hospital, in Cape Coast, Ghana. Additional samples were obtained from staff and students at the West African Center for Cell Biology of Infectious Pathogens, University of Ghana (WACCBIP). Individuals with active SARS-CoV-2 infection were identified based on quantitative reverse transcription PCR (qRT-PCR) results, written informed consent was obtained, and blood samples were collected. Blood samples were processed into plasma and peripheral blood mononuclear cells (PBMCs).

PBMC samples were collected from 217 patients with COVID-19 disease; of these, $n = 82$ individuals who consented to follow-up sampling were sampled every week for four weeks to allow for the characterization of immune responses over time.

Symptomatic and asymptomatic participants were categorised according to WHO definitions (WHO 2020).Thus, symptomatic participants were defined as those who tested positive for SARS-CoV-2 with COVID-19 symptoms such as fever or chills, cough, shortness of breath or difficulty breathing, fatigue, muscle or body aches, and loss of taste or smell, while asymptomatic cases were defined as individuals who tested positive for SARS-CoV-2 but had no reported COVID-19 symptoms. The patient's disease status was taken from medical reports and/or through a questionnaire.

METHOD DETAILS

Ethics approval and consent to participate

The study protocol was reviewed and approved by the Ethics Board of the College of Basic and Applied Sciences, University of Ghana (ECBAS 063/19–20), and the Ethical Review Committee of the Ghana Health Service (GHS-ERC 011/03/20). Additional formal written permissions were obtained from sample collection sites. Participation was voluntary and written informed consent was obtained from all participants before enrollment.

Sample collection and preparation

Peripheral blood (5 mL) was collected into ethylenediamine tetraacetic acid (EDTA) tubes using the venepuncture method. Baseline samples were collected immediately after PCR confirmation of SARS-CoV-2 infection, or concurrently with sample acquisition for PCR, then included in the study once a positive result had been obtained.

PBMC isolation, cryopreservation, and thawing

PBMCs were isolated from whole blood using density gradient centrifugation as previously described with minor modifications (Marchi et al. 2021). Briefly, whole blood was centrifuged at 2,500 rpm for 5 min to separate plasma from blood cells. Upon removal of the plasma fraction which was kept in a −80°C freezer, the cellular fraction was subjected to Ficoll density gradient centrifugation at 2500 rpm for 20 min. The PBMCs were washed and resuspended in a freezing medium prepared with 90% fetal bovine serum (FBS) and 10% dimethyl sulfoxide (DMSO) before freezing at −80°C and later transferred to liquid nitrogen for storage. For use, frozen PBMCs were quick thawed in a water bath (37°C), transferred to a pre-warmed cell culture medium (RPMI with 10% FBS) with benzonase (25U/mL), and rested for 1 h at 37°C in 5% of CO2.

Culture of malaria parasites and isolation of merozoites

Erythrocytic stages of *P. falciparum*-DD2 strain were cultured as previously described (Deshpande and Shastry 2004) and merozoites were isolated following protocol from Quadt et al. (2020) with some modifications. Briefly, 0.1% saponin prepared in PBS (1X) was added into cultured red blood cells containing parasites and incubated on ice for 10 min to reduce exothermic reaction. The lysate was centrifuged, and the pellet (merozoites) was washed with PBS (1X). The pellets were stored at −20°C waiting for analysis.

Immunophenotyping

The rested PBMCs (100μL) were stained with a cocktail of antibodies (Abs) and brilliant stain buffer (BD Biosciences) in a 100μL staining volume and mixed. The cocktail had fluorochrome-conjugated anti-human Abs against CD3-BUV 395, CD8-APC H7, CD4-PE, CD19-BV 785, CD21-BB 515, CD27-PE/Cy7, CD14-APC, CD16-PerCP/cy5.5, PD-1-BUV 737, CD38-BV 605, HLA-DR-BV 510, and Alexa Fluor 700 (all from BioLegend, Inc.). The mixture was incubated in the dark at room temperature for 30 min. The cells were then washed twice with FACS buffer (2% BSA in PBS(1X)) and centrifuged at 3000rpm for 3 min. The pelleted cells were resuspended in 300uL of PBS(1X) and 300,000 events were acquired in a flow cytometer (BD Biosciences, BD LSR Fortessa TM X-20 Special Order System). The data were analyzed using FlowJo v10.8 Software (BD Life Sciences) and cell gating strategy is shown in Figure S1.

In vitro stimulation and intracellular cytokine staining

To investigate the functionality of T cells, their cytokine production was assessed upon stimulation in a cell culture system. Thawed PBMCs were seeded at 2 million cells per well in a 96-well round bottom plate, in a total volume of 100 μL of cell culture medium (RPMI 1640 (Gibco), supplemented with 10% FBS (Gibco) and 1% penicillin-streptomycin (Gibco)). The cells were stimulated in total of 19 h at 37°C in 5% CO2 using wild type SARS-CoV-2 Spike (Ab 273063, Abcam) and Nucleocapsid (Ab 273530, Abcam), and P. falciparum lysate (DD2 strain) all at 10 μg/mL. Phytohaemagglutinin (PHA) was used as a positive control and unstimulated cells were used

as background control. Co-stimulatory antibodies (CD49d and CD28) were also added to the assay wells. PBMCs were first incubated for 2hours at 37°C in 5% CO2 and protein transport inhibitors, brefeldin A (Golgi Plug, Becton Dickinson) and monensin (Golgi stop, Becton Dickinson) were added at 5μg/mL each, and incubated further for 17 h.

After stimulation, cells were washed and surface stained with fluorochrome-conjugated anti-human Abs against CD3-BUV 395, CD8-APC-H7, and CD4-PE (BioLegend, Inc.) for 30 min on ice. Cells were then washed with FACS buffer, fixed and permeabilized with fixation/permeabilization buffer set (eBioscience, Inc.) on ice for 30 min following manufacturer's protocol. Intracellular staining with Abs recognizing IL-10-PE/Cy7, IL-17A-BV 510, IL-21 BV-421, TNF-α-FITC and IFN-γ-BV 786 (all from BioLegend, Inc.) was done for 30 min on ice. The cells were then washed and resuspended in PBS(1X) and a minimum of 100,000 events were acquired on a flow cytometer (BD Biosciences, BD LSR Fortessa TM X-20 Special Order System). The data obtained were analyzed using FlowJo v10.8 Software (BD Life Sciences). Lymphocytes were gated using morphological parameters according to their forward and side scatter characteristics. The labeling antibody scheme was used further to place cytokine gates around $CD3^+$ and $CD4^+$ T cells as well as $CD3^+$ and $CD8^+$ T cells (Figure S2). Boolean gating approach was used to identify the percentages of $CD4^+$ and $CD8^+$ T cells producing single or multiple cytokines.

The cytokine levels used in the correlation analysis were adapted from our previous study which was conducted on the same patients. These cytokines were measured from plasma samples and quantified using Multiplex Luminex assay as described.[33]

Detection of anti-*Plasmodium falciparum* antibodies

An in-house indirect enzyme-linked immunosorbent assay (ELISA) was used to detect antibodies directed against some surface antigens of *P. falciparum* in plasma samples collected from study participants. The antigens used *Plasmodium falciparum* circumsporozoite Protein (PfCSP), PF3D7_1410700 (E−14), PF3D7_0507400 (E−17), Reticulocyte binding-like protein homologue 5 (RH5) and merozoites-associated armadillo repeats protein (PfMAAP). The antigens were codon-optimized and cloned into pET9b plamid for expression in BL21 bacteria cells, except for PfMAAP which was codon-optimized and cloned into Expression vector pcDNA3.4 (Genscript) for expressed in mammalian cell. The antigen were expressed and purified to homogeneity (98% purity). The antigens were stored in 20mM Tris, 150mM NaCl at pH 7.5, supplemented with 0.5% glycerol and protease inhibitor cocktail. These antigens are primarily found in the blood stage, which is the most infectious stage of the malaria parasite. The presence of antibodies against these antigens may indicate recent exposure. They play a crucial role in diagnosing malaria and are potential candidates for a vaccine.

Briefly, Nunc Maxisorp plates were coated with 50μL of 2μg/mL antigen overnight at 4°C, followed by blocking with 200μL per well of 3% bovine serum albumin (BSA) at room temperature for 1 h. A dilution of 1:1000, of plasma samples and calibration standards were added at 50μL per well in duplicates and incubated for 2 h at room temperature in the dark. The standard curve was generated using a 1:200 dilution of pooled sera from malaria-exposed individuals diluted 2-fold across 7 pairs of wells. The secondary antibody, goat anti-human IgG conjugated to HRP (seracare, 5220-0330) was diluted at 1:10,000 and 50μL was added in each well and incubated in the dark at room temperature for 1 h. After each stage of the process, the plate was washed 6 times with washing buffer and thoroughly dried. The color reaction was generated using 100μL of 3,3′,5,5′-Tetramethylbenzidine for 2 min and stopped with 0.27N H2SO4. The plates were read using the Varioskan ELISA plate reader (Thermo Scientific) at 450nm wavelength. Optical densities were converted to concentrations (arbitrary units) using Auditable Data Analysis and Management System for ELISA Version 1.1 (ADAMSEL FPL).

QUANTIFICATION AND STATISTICAL ANALYSIS

Raw data was saved in Excel format and data analysis was performed using GraphPad Prism Version 9 (GraphPad Software, San Diego, California USA). Since data were not normally distributed, immune parameters between symptomatic and asymptomatic groups were compared using the Mann-Whitney U test. Multiple comparisons between different groups were tested using Kruskal-Wallis with Dunn's post hoc test with no correction (ns: $p > 0.05$, *: $p < 0.05$, **: $p < 0.01$, ****: $p < 0.0001$).

Cell Reports, Volume *43*

Supplemental information

Cellular immune response to SARS-CoV-2 and clinical presentation in individuals exposed to endemic malaria

Kesego Tapela, Diana Ahu Prah, Becky Tetteh, Franklin Nuokpem, Daniel Dosoo, Amin Coker, Frederick Kumi-Ansah, Emmanuella Amoako, Kissi Ohene Assah, Charlyne Kilba, Nancy Nyakoe, Darius Quansah, Sylvester Languon, Claudia Adzo Anyigba, Felix Ansah, Seth Agyeman, Irene Amoakoh Owusu, Kristan Schneider, William K. Ampofo, Joe Kimanthi Mutungi, Gloria Amegatcher, Yaw Aniweh, Gordon A. Awandare, Peter K. Quashie, and Yaw Bediako

Supplementary figures

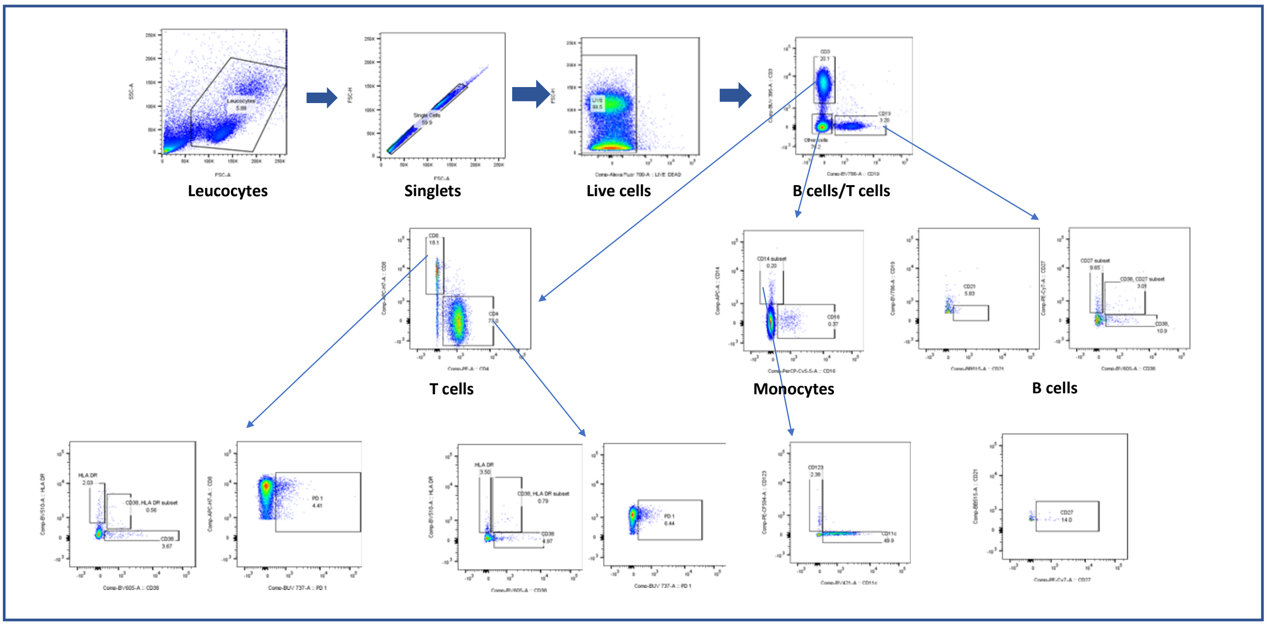

Figure S1: Cell gating for immunophenotyping, Related to Figure 1

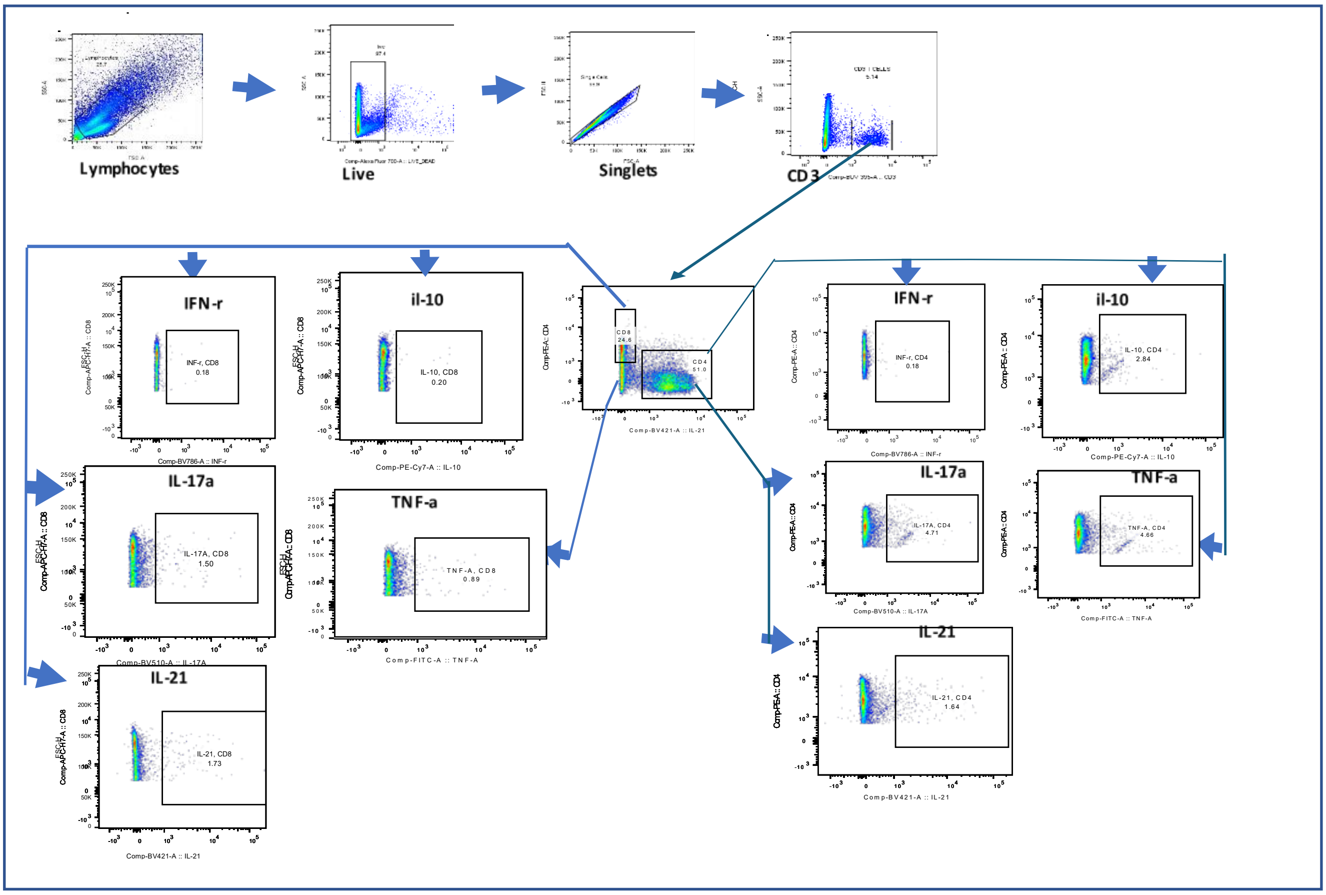

Figure S2: Cell gating for intracellular staining, Related to Figure 2.

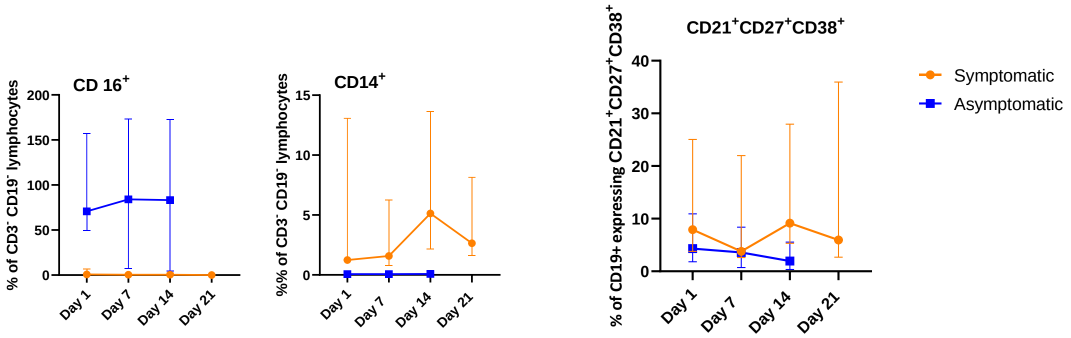

Figure S3: The change in surface cell frequencies over time, Related to Figure 1. The percentage cell proportions were analysed from PBMC of COVID-19 symptomatic (n = 53) and asymptomatic (n =29) patients at different time points for four weeks. The median quantity of the percentage cell proportions for each sampling day is shown by the line graph and the 25th and 75th percentiles are cell percentage proportions.

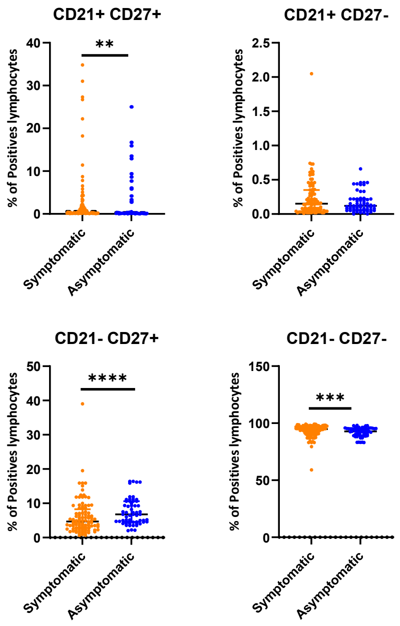

Figure S4: The scatter plots showing the proportion of classical memory B cells ($CD21^{+}CD27^{+}$), naïve B cells ($CD21^{+}CD27^{-}$), atypical memory B cells ($CD21^{-}CD27^{-}$) and activated memory B cells ($CD21^{-}CD27^{+}$), Related to Figure 1. A horizontal line across the scatter plots shows the median baseline samples per participant while the lower and upper dotted lines represent the 1st and 3rd percentiles, respectively. Statistical significance between symptomatic and asymptomatic patients were determined by a two-tailed Mann-Whitney U test (ns: $p > 0.05$, *: $p < 0.05$, **: $p < 0.01$, ****: $p < 0.0001$).

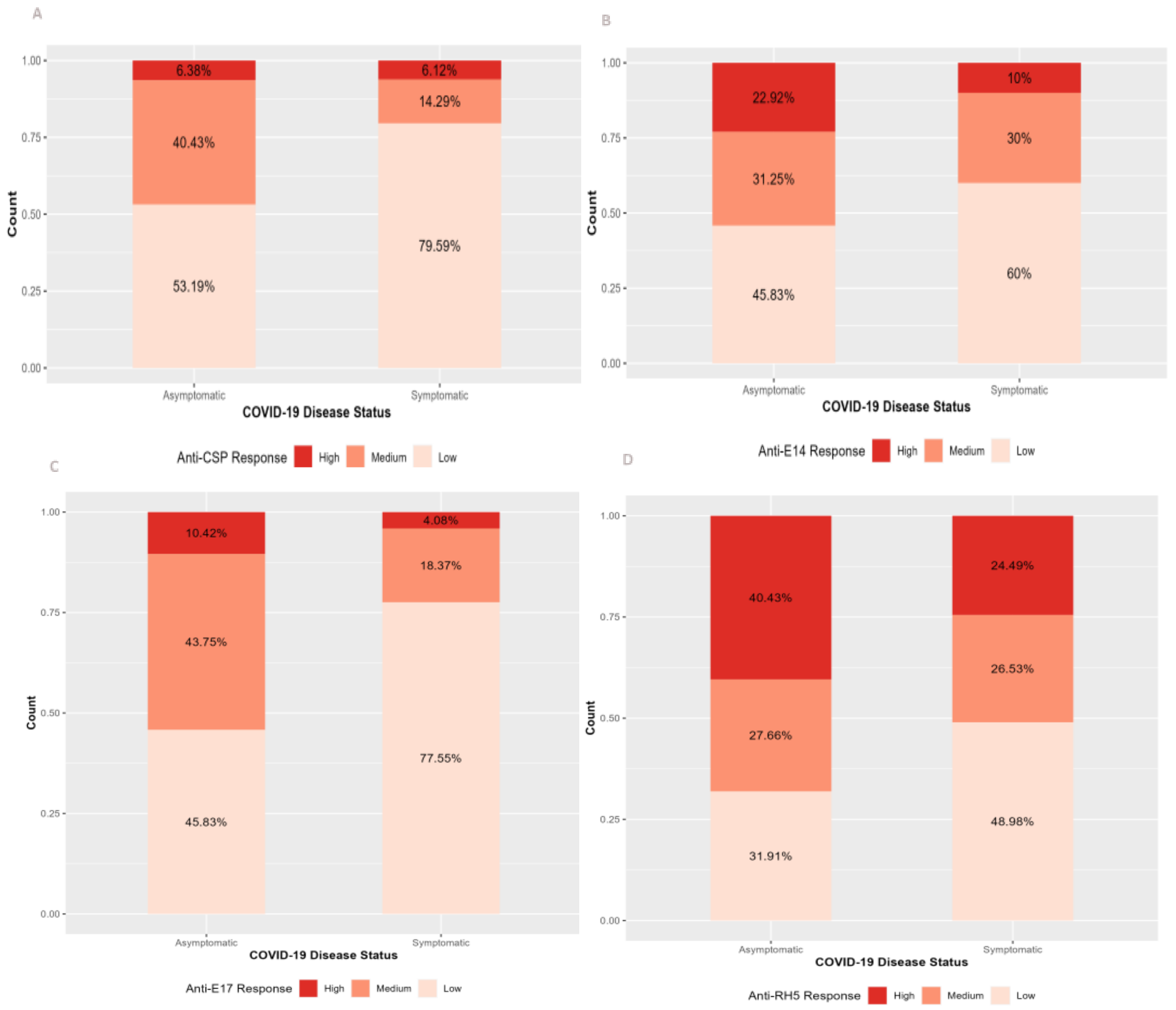

Figure S5: **The stacked bar charts comparing the proportions of antibody levels from (A):PfCSP ,(B):E-14, (C):E-17 and (D):RH5 in symptomatic(n=50) and asymptomatic(n=50) patients, Related to Figure 3.** A finite mixture model was used to segregate the dataset into subpopulations; low, medium, and high antibody responses.

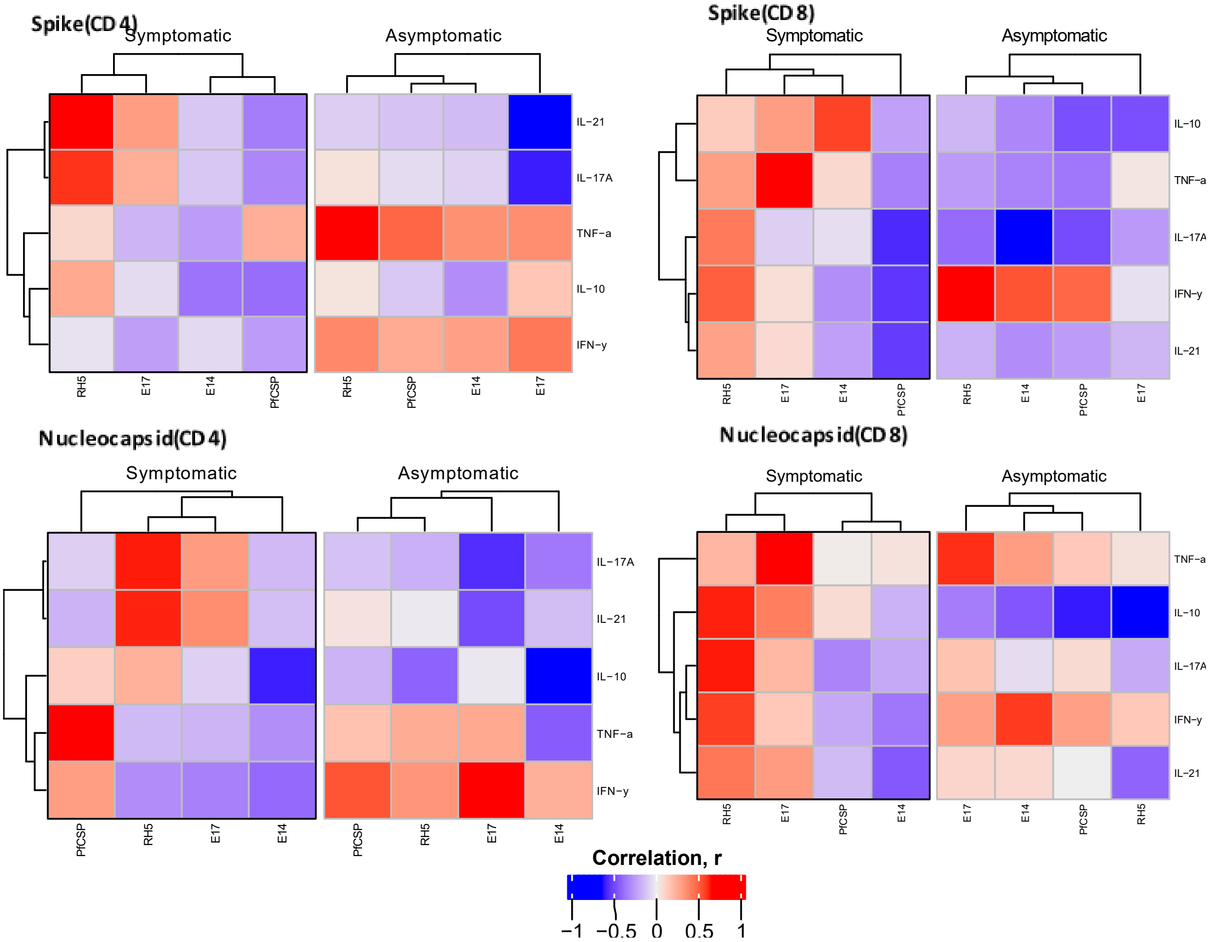

Figure S6:Correlations between anti-*P.falciparum* antibodies and cytokines in symptomatic and asymptomatic COVID-19 individuals, Related to Figures 2 & 3. The cytokine-antibodies clustering patterns are shown in heatmaps and the magnitude of correlation coefficients is shown on a color scale of red (strong positive correlation), white (no correlation) to dark blue (strong negative correlation) signal.

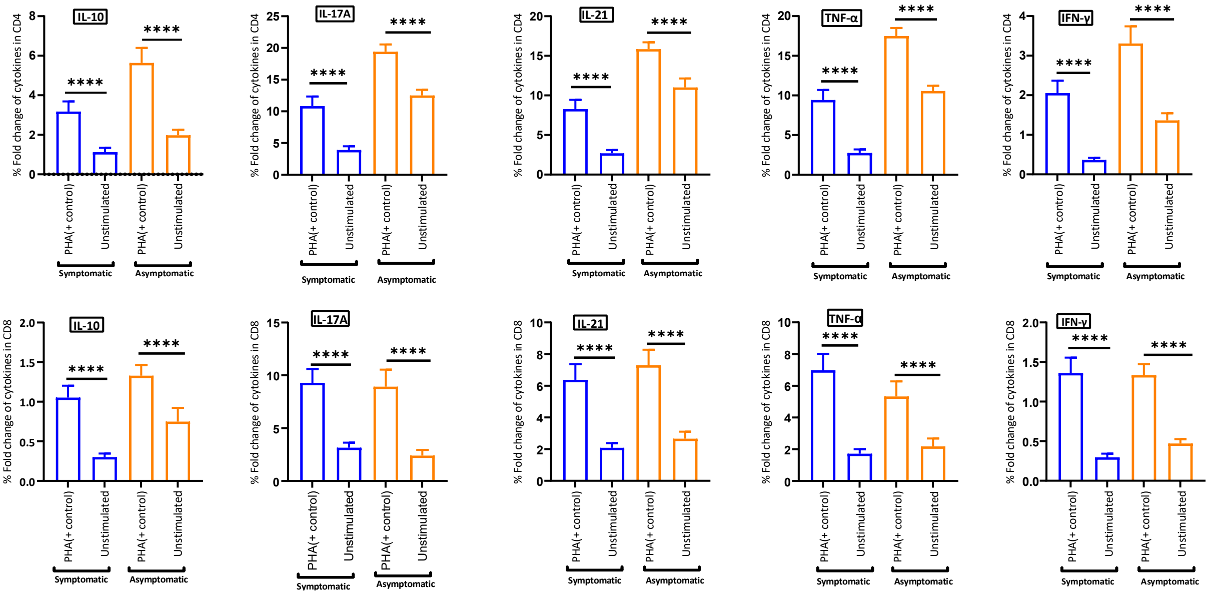

Figure S7: **Comparison of percentage stimulation of cytokines by PHA and unstimulated in symptomatic (n=116) and asymptomatic (n=44) COVID-19 patients, Related to Figure 2.** The bar plots show mean with standard error bars. The percentages were calculated by subtracting the percentages of stimulated from unstimulated cells. Statistical significance was determined by Mann-Whitney U test (ns: p >0.05, *: p <0.05, **: p < 0.01, ****: p < 0.0001).
